# SUMOylation-related genes define prognostic subtypes in stomach adenocarcinoma: integrating single-cell analysis and machine learning analyses

**DOI:** 10.3389/fimmu.2025.1527233

**Published:** 2025-08-01

**Authors:** Kaiping Luo, Donghui Xing, Xiang He, Yixin Zhai, Yanan Jiang, Hongjie Zhan, Zhigang Zhao

**Affiliations:** ^1^ Department of Medical Oncology, Tianjin First Central Hospital, School of Medicine, Nankai University, Tianjin, China; ^2^ Department of Oncology, Tianjin Medical University Cancer Institute and Hospital, National Clinical Research Center for Cancer, Key Laboratory of Cancer Prevention and Therapy, Tianjin’s Clinical Research Center for Cancer, Tianjin, China; ^3^ Department of Gastroenterology, Tianjin Medical University Cancer Institute and Hospital, National Clinical Research Center for Cancer, Key Laboratory of Cancer Prevention and Therapy, Tianjin’s Clinical Research Center for Cancer, Tianjin, China

**Keywords:** stomach adenocarcinoma, sumoylation, machine learning, L3MBTL2, VHL

## Abstract

**Background:**

Stomach adenocarcinoma (STAD) exhibits high molecular heterogeneity and poor prognosis, necessitating robust biomarkers for risk stratification. While SUMOylation, a post-translational modification, regulates tumor progression, its prognostic and immunological roles in STAD remain underexplored.

**Methods:**

Prognostic SUMOylation-related genes (SRGs) were screened via univariate Cox regression, and patients were stratified into molecular subtypes using unsupervised consensus clustering. A SUMOylation Risk Score (SRS) model was developed using 69 machine learning models across 10 algorithms, with performance evaluated by C-index and AUC. Immune infiltration, pathway enrichment identified key SRGs, and *in vitro* functional assays were validated.

**Results:**

Two molecular subtypes (A/B) with distinct SUMOylation patterns, survival outcomes (log-rank *p* < 0.001), and immune microenvironments were identified. The random survival forest (RSF)-based SRS model (AUC: 0.97) stratified patients into high-/low-risk groups, where high-risk patients exhibited advanced tumor stages, immune suppression, and elevated TIDE scores (*p* < 0.001). Functional enrichment linked low-risk groups to genome stability pathways (DNA repair, cell cycle control). *In vitro* validation confirmed that L3MBTL2 and VHL knockdown promoted proliferation, migration, and invasion in AGS cells (*p* < 0.05).

**Conclusion:**

This study establishes SRGs as independent prognostic indicators and defines SUMOylation-driven subtypes with distinct immune and molecular features. The SRS model and functional validation of L3MBTL2/VHL provide actionable insights for personalized STAD management and immunotherapy targeting. (214 words)

## Highlights

- A machine learning-derived SUMOylation Risk Score (SRS) model achieved high predictive accuracy (AUC = 0.97) and stratified patients into clinically distinct risk groups.
- Functional validation revealed tumor-suppressive roles of L3MBTL2 and VHL, highlighting their potential as therapeutic targets in STAD.

## 1 Introduction

Gastric cancer (GC) is the fifth most common cancer and the fourth leading cause of cancer-related death worldwide (1, 2). Stomach adenocarcinoma (STAD), arising from the glandular epithelium of the stomach, represents the predominant histological subtype and is commonly divided into intestinal and diffuse forms. Multiple factors such as chronic infection with *Helicobacter pylori*, host susceptibility, dietary factors and environmental exposures contribute to STAD pathogenesis (3–5). Due to its often asymptomatic nature, many patients are diagnosed at advanced stages, resulting in a poor prognosis (6, 7). These challenges highlight the urgent need to identify novel prognostic biomarkers to support early detection and personalized therapy.

SUMOylation is a reversible post-translational modification (PTM) mediated by small ubiquitin-like modifiers (SUMOs), which regulate critical cellular processes including DNA repair, cell division, and programmed cell death (8–11). Despite increasing interest in SUMOylation across various cancers such as breast, colorectal and hematologic malignancies (12–14), systematic investigations of SUMOylation-related genes (SRGs) and their prognostic relevance in STAD are still lacking.

The advent of next-generation sequencing technologies, particularly single-cell RNA sequencing (scRNA-seq) and bulk RNA sequencing, has transformed our understanding of tumor heterogeneity and immune landscapes (15–17). Specific cell populations within the tumor microenvironment serve as prognostic indicators. For instance, myeloid-derived suppressor cells (MDSCs) facilitate tumor immune evasion, increasing the likelihood of poor outcomes, whereas CD8^+^ T cells are associated with improved clinical outcomes (18–20). By characterizing the composition of tumor-infiltrating immune cells, we can better understand the progression and develop immunologically relevant prognostic tools in various cancers (21).

With the increasing complexity of omics data, machine learning (ML) approaches are becoming indispensable for deciphering high-dimensional datasets and identifying disease-relevant molecular features (22–24). Using multiple ML algorithms enables more robust feature selection and prediction by minimizing model-specific limitations (25, 26). In this study, we constructed 69 predictive models across 10 machine learning algorithms to evaluate the prognostic utility of SRGs.

By integrating single-cell and bulk transcriptomic data with machine learning, we systematically identified prognostic SRGs and stratified STAD patients into distinct molecular subtypes. Our findings reveal two subtypes with differing survival outcomes and immune profiles, and suggest that genes such as L3MBTL2 and VHL may act as protective biomarkers. This study offers new insight into the involvement of SUMOylation in STAD and proposes candidate genes for prognostic evaluation and therapeutic development. (377 words)

## 2 Methods

### 2.1 Data collection and integration

Single-cell RNA sequencing (scRNA-seq) data for stomach adenocarcinoma (STAD) were obtained from the TISCH database (tisch.comp-genomics.org, accession number GSE167297), encompassing 18,351 genes across 22,464 cells. Bulk RNA-seq data were downloaded from The Cancer Genome Atlas (TCGA-STAD, https://portal.gdc.cancer.gov/) with 375 patient samples, and from the Gene Expression Omnibus (GEO, GSE62254, https://www.ncbi.nlm.nih.gov/geo/) with 300 patient samples. After merging TCGA-STAD and GSE62254 datasets, a combined cohort of 654 STAD samples containing 16,928 common genes was constructed. Batch effects resulting from dataset integration were corrected using the ComBat function from the “sva” R package, which applies an empirical Bayes framework to remove technical variation while preserving biological signals.

### 2.2 Single-cell transcriptomics and functional pathway analysis

The STAD scRNA-seq dataset (GSE167297) was processed using the “Seurat” R package for normalization, dimensionality reduction, and clustering. Cell type annotations were obtained from the TISCH database (27). Marker genes for each cell type were identified using the “Cell-type Orientation Scoring for Genes (COSG)” package, and differentially expressed genes (DEGs) were filtered with the “FindAllMarkers” function (28). The top 5 marker genes per cell type were visualized via the “DoHeatmap” and “scRNAtoolVis” packages.

Gene sets including Hallmark, Biocarta, Kyoto Encyclopedia of Genes and Genomes (KEGG), Reactome, and WikiPathway were retrieved from the MSigDB database (29). Enrichment analyses were performed using the “clusterProfiler” package based on the top 100 marker genes per cell type identified by “COSG”. Pathway activity scores were calculated by “GSVA” package and visualized with “pheatmap” to depict subtype-specific pathway activation patterns.

### 2.3 Expression and scoring of SUMOylation-related genes

A set of 200 SUMOylation-related genes (SRGs) was compiled by merging gene lists from a published lung adenocarcinoma study and the Reactome pathway database (30). Differential expression analysis of SRGs in STAD was performed using the “limma” package, applying thresholds of fold change ≥ 2 and false discovery rate (FDR) < 0.05 for statistical significance (31). Single-cell data preprocessing, dimensionality reduction, and clustering were conducted with “Seurat” to facilitate cell type identification and UMAP visualization. SUMOylation pathway activity per cell was assessed using the “GSVA” package, generating SUMOylation scores based on SRGs expression (30). UMAP plots were used to visualize clusters stratified by high and low SUMOylation scores.

### 2.4 Identification of SRGs in STAD

From the merged dataset, 200 SRGs were analyzed, with 179 genes were effectively represented in the expression profile. Correlation analyses and univariate Cox regression were conducted via the “survival” and “survminer” packages, identifying 42 genes significantly associated with prognosis (Cox *p* < 0.001). Unsupervised clustering was performed with “ConsensusClusterPlus” package, which classified the samples into two subtypes (A and B) (32). Survival differences between subtypes were evaluated, and differential expression analyses highlighted subtype-specific gene expression patterns. A heatmap generated by the “pheatmap” package depicted the relationships between SRG expression and subtypes.

### 2.5 Immune microenvironment analysis and visualization

Principal component analysis (PCA) was performed via the “prcomp” function from the “stats” package to visualize the sample distribution. Immune cell infiltration differences between subtypes were evaluated by calculating infiltration scores via single-sample gene set enrichment analysis (ssGSEA) implemented in the “GSVA” package. To comprehensively assess immune infiltration within the tumor microenvironment (TME), eight deconvolution methods from the “IOBR” package-MCPcounter, EPIC, xCell, CIBERSORT, IPS, quanTIseq, ESTIMATE, and TIMER- were applied (33). Heatmaps were generated to illustrate the relationships between subtypes and immune cells within the tumor microenvironment (TME).

### 2.6 Machine learning-based risk signature construction

The TCGA-STAD dataset was utilized as the training set, whereas GSE62254 dataset served as the validation set. Ten widely used machine learning algorithms were employed to construct prognostic risk models, including Random Survival Forest (RSF), Least Absolute Shrinkage and Selection Operator (LASSO), Gradient Boosting Machine (GBM), Survival Support Vector Machine (Survival-SVM), Supervised Principal Components (SuperPC), Ridge Regression, Partial Least Squares Regression for Cox (plsRcox), CoxBoost, Stepwise Cox Regression, and Elastic Network (Enet) (33). Risk score for each dataset was calculated via the following formula: risk score = Σ (coefficient × expression) (34). Among these algorithms, RSF, LASSO, CoxBoost, and stepwise Cox were specifically used for dimensionality reduction and variable selection. Multiple algorithmic combinations totaling 69 approaches were tested, with the optimal model selected based on the highest average concordance index (C-index) (35). The final SUMOylation Risk Score (SRS) model quantifies the prognostic impact of SRGs in STAD.

### 2.7 Evaluation of model performance

Using the best-performing machine learning model, samples were dichotomized into high- and low-risk groups by the median risk score in training, validation, and combined cohorts. Receiver operating characteristic (ROC) curves and area under the curve (AUC) metrics assessed predictive accuracy. Time-dependent ROC analyses further evaluated model performance over time (36). Kaplan–Meier survival analysis and log-rank tests compared overall survival (OS) between risk groups. Univariate and multivariate Cox regression analyses verified the independence of the risk signature. Expression of key genes identified by the random forest model was visualized in single cells using UMAP plots.

### 2.8 Drug sensitivity and immunotherapy response prediction

Mutation profiles of the high- and low-risk groups were analyzed via the “maftools” package, and mutation patterns were visualized via a waterfall plot. Differences in gene mutation frequencies were compared, and associations with Tumor Immune Dysfunction and Exclusion (TIDE), EXCLUSION, and DYSFUNCTION scores were evaluated (37). Additionally, variations in the mRNA stemness index (mRNAsi), microsatellite instability (MSI), and tumor mutational burden (TMB) were analyzed to predict immune evasion, recurrence risk, and response to immunotherapy. Drug sensitivity between the two groups was assessed via the “pRRophetic” package (38).

### 2.9 Cell culture and transfection

The human STAD cells were maintained in RPMI-1640 medium (Gibco, USA) supplemented with 10% fetal bovine serum (NEWZERUM, China) and 1% penicillin-streptomycin under standard conditions (37°C, 5% CO_2_). To achieve gene knockout of L3MBTL2 and VHL (Von Hippel-Lindau), shRNA plasmids (Genechem, Shanghai, China) were transiently transfected into AGS cells using polyethyleneimine (PEI)-mediated transfection. AGS cells were seeded in 6-well plates at a density of 2.5 × 10^6^ cells per well and incubated overnight until reaching approximately 80% confluence.

For each well, 12 µg plasmid was diluted in 100 µL Opti-MEM (Gibco), and PEI (1 mg/mL, 3:1 PEI: DNA ratio) was diluted in another 100 µL Opti-MEM. After 5 minutes of separate incubation, the DNA and PEI mixtures were combined and incubated for 15 minutes at room temperature. The mixture was then added dropwise to the cells. After 6–8 hours of incubation, the transfection medium was replaced with fresh RPMI-1640 medium containing 10% FBS and 1% penicillin-streptomycin. Seventy-two hours post-transfection, cells were collected for western blot analysis.

### 2.10 Western blot analysis

Total protein was extracted from AGS cells by lysing cell pellets in a mixture of RIPA buffer, 10× protease inhibitor cocktail, and 100× PMSF (all from Beyotime, China) on ice for 30 minutes. The lysates were then sonicated and centrifuged at 4°C to remove cell debris. The supernatants were collected into fresh EP tubes, and protein concentrations were determined using the BCA Protein Assay Kit (Beyotime, China) according to the manufacturer’s protocol. The optical density (OD) at 562 nm was measured using a multimode plate reader (PerkinElmer, USA).

Equal amounts of protein were separated by 10% SDS-PAGE, initially running at 80 V for 30 minutes, followed by 120 V for 60 minutes. Proteins were transferred to PVDF membranes (IMMOBILON, IPVJ00010) using the Bio-Rad wet transfer system at a constant current of 250 mA for either 40 minutes (VHL) or 90 minutes (L3MBTL2). Membranes were blocked with 5% non-fat milk in TBST for 90 minutes at room temperature and incubated overnight at 4°C with the following primary antibodies: anti-L3MBTL2 (rabbit polyclonal, ABclonal, A10331, 1:1000), anti-VHL (rabbit monoclonal, ABclonal, A23239, 1:1000), and anti-β-actin (ABclonal, AC026, 1:5000) as a loading control. After incubation with HRP-conjugated secondary antibodies (ProteinTech, Cat No:81115-1-RR, 1:5000), protein bands were visualized using an ECL detection system.

### 2.11 Cell proliferation assay

Post-transfection, AGS cells were seeded at a concentration of 3,000 cells per well. Cell viability was measured at 0, 24 and 48 hours using the Cell Counting Kit-8 (CCK-8; Dojindo, Japan) following manufacturer instructions. Absorbance at 450 nm was measured to evaluate proliferation using a microplate spectrophotometer (BioTek, USA).

### 2.12 Transwell migration and invasion assay

Transwell chambers (8 μm pore size, 24-well format; Corning, USA) were used to evaluate the migration and invasion abilities of AGS cells. For invasion assay, upper chambers were pre-coated with a mixture of Matrigel and serum-free medium at a ratio of 1:7, and incubated at 37 °C overnight to allow gel formation. No coating was applied for the migration assay.

AGS cells were harvested and resuspended in serum-free medium. A total of 1 × 10^5^ cells were seeded into the upper chambers (coated or uncoated), while the lower chambers were filled with complete medium containing 10% FBS medium. After 12 hours of incubation, non-migrated or non-invaded cells were removed from the upper membrane surface. Cells that had passed through the membrane were fixed with anhydrous methanol for 20 minutes, stained with 0.1% crystal violet for 20 minutes, and visualized using a light microscope at 4× magnification. The number of cells was counted in three randomly selected fields per well. All experiments were performed in triplicate, and quantitative results were expressed as mean ± SD.

### 2.13 Wound healing scratch assay

AGS cells were plated into 6-well plates at a density of 5 × 10^5^ cells per well and incubated overnight at 37°C in a humidified incubator with 5% CO_2_. Upon reaching approximately 90% confluence, a linear scratch was generated across the cell monolayer using a sterile 200 μL pipette tip under uniform pressure. Detached cells were carefully removed by washing twice with phosphate-buffered saline (PBS), followed by incubation in serum-free medium to minimize the influence of cell proliferation. Phase-contrast images of the wound area were acquired at 0, 12, and 24 hours using an inverted microscope at consistent magnification. The extent of cell migration was evaluated by quantifying the wound area using ImageJ software (version 1.54p, NIH, USA), and the wound closure rate was calculated using the following formula:


$$Wound\;closure\left(\%\right)=\left(Area_{0h}-Area_{12h}\right)/Area_{0h}\times 100\%$$

### 2.14 Statistical analysis

Kaplan–Meier curves and the log-rank test were applied to assess survival differences between risk groups. Correlations between continuous variables were determined using Pearson’s correlation analysis. The Wilcoxon rank-sum test was used to compare differences in non-normally distributed data. All analyses and data visualization were carried out using R software (version 4.2.2), with a two-tailed *p*-value < 0.05 considered statistically significant. Microscopic image analysis was performed using ImageJ (version 1.54p, NIH, USA).

## 3 Results

### 3.1 Single-cell landscape reveals cellular heterogeneity and activation of immune-associated pathways in STAD

**Figure 1:**
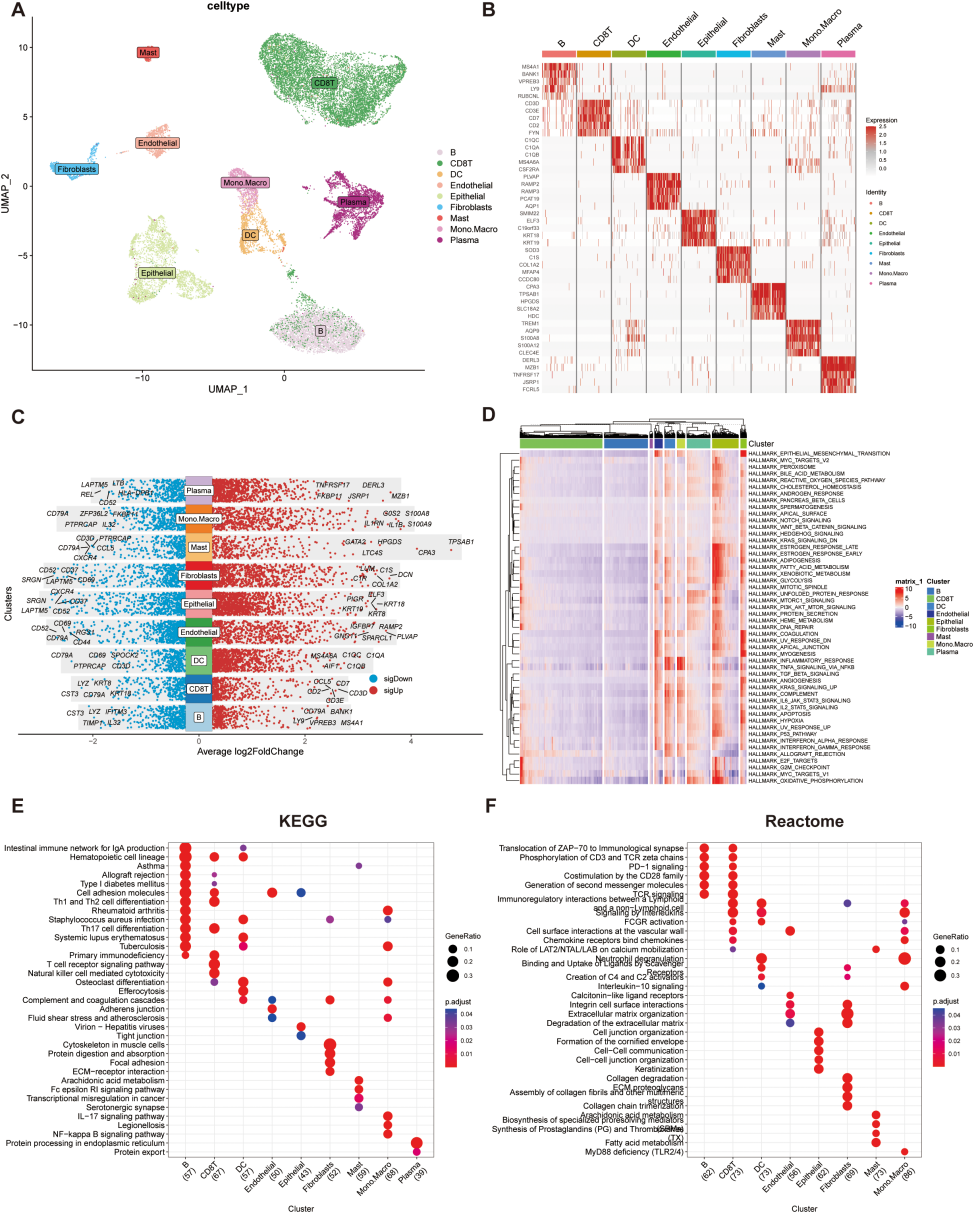
Single-cell RNA sequencing (scRNA-seq) data analysis integrating TISCH and MsigDB databases. **(A)** UMAP visualization of cell subpopulation. Clusters were annotated based on the expression of canonical cell marker genes. **(B)** Heatmap showing the top five differentially expressed genes (DEGs) for each cell cluster. **(C)** Dot plot illustrating the top five upregulated and downregulated genes within each cell cluster. Gene expression levels are represented by color intensity. **(D)**Heatmap depicting pathway enrichment scores for individual cell clusters across 50 hallmark pathways from the MSigDB database. **(E)** KEGG pathway enrichment analysis of the top 100 cluster-specific genes identified by COSG. Significantly enriched pathways are highlighted. **(F)** Reactome pathway enrichment analysis of the top 100 cluster-specific genes identified by COSG.

Nine distinct cell clusters were identified via dimensionality reduction and clustering, including B cells, CD8^+^ T cells, dendritic cells (DCs), endothelial cells, epithelial cells, fibroblasts, mast cells, monocyte-macrophages, and plasma cells (**Figure 1A**).

The top five marker genes for each cluster were selected and visualized in a heatmap to highlight cluster-specific gene expression patterns (**Figure 1B**), with further refinement confirming the most and least expressed genes unique to each cell type (**Figure 1C**).

Pathway activity analysis across the nine cell types revealed that epithelial cells exhibited significantly elevated activity in proliferation, cell cycle regulation, and energy metabolism pathways, notably enriched in E2F targets, G2M checkpoint, and MYC targets (**Figure 1D**). Functional enrichment of the top 100 genes per cell type showed distinct pathway profiles in KEGG and Reactome databases (**Figures 1E, F**). B cells and CD8^+^ T cells were enriched in pathways related to cell adhesion molecules and Th1/Th2 cell differentiation. Reactome analysis highlighted their involvement in immune signaling processes, including the translocation of ZAP-70 to the immunological synapse, CD3 and TCR zeta chain phosphorylation, and PD-1 signaling.

### 3.2 High SUMOylation activity correlates with B cell reduction and activation of proliferation pathways in STAD

**Figure 2:**
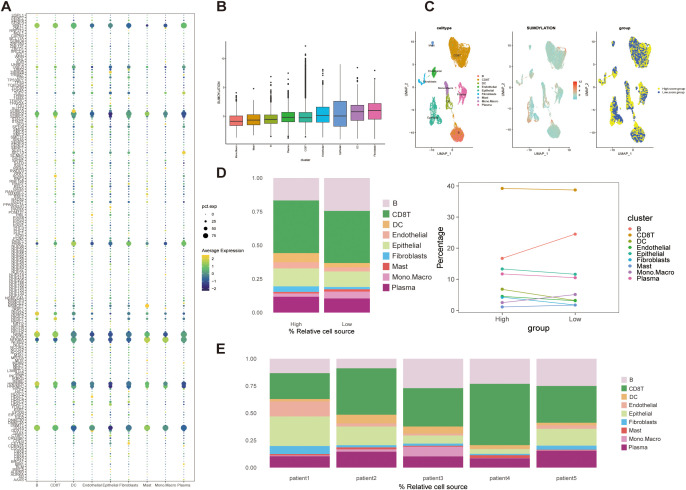
Expression patterns and GSVA scoring of SUMOylation-related genes (SRGs) in STAD single-cell data. **(A)** Bubble plot showing SRGs expression across nine cell types. **(B)** Cellular-level SUMOylation activity scores, illustrating the distribution of scores across individual cells. **(C)** UMAP visualization of cell clustering based on median SUMOylation activity scores. Cells were categorized into high- and low-score groups. **(D)** Cell composition analysis. Left: Proportions of cells stratified by high and low SUMOylation activity scores. Right: Trends in cell type distribution between high- and low-scoring groups. **(E)** Patient-specific cell composition, highlighting the proportion of cells with high and low SUMOylation activity scores.

SUMOylation-related genes (SRGs) showed distinct expression patterns across the nine cell populations, with GSVA-based scoring revealing variable SUMOylation activity levels (**Figures 2A, B**). Based on the median SRGs scores, all the cells were categorized into high- and low-score groups, and their spatial distributions was visualized using a UMAP plot (**Figure 2C**).

Notably, compared the cell composition between the two groups, B cells were significantly enriched in the low-score group, whereas the populations of other cell types exhibited only minor variations (**Figure 2D**). This pattern was validated in bulk patient datasets, which confirmed the predominance of CD8^+^ T cells and relatively low mast cell abundance, consistent with single-cell observations (**Figure 2E**). The strong alignment between single-cell and bulk data emphasizes that B cells and CD8^+^ T cells are major immune components within the STAD microenvironment. Notably, although CD8^+^ T cells remained abundant regardless of SUMOylation status, B cells significantly decreased in the high SUMOylation group, suggesting a specific association between SUMOylation activity and B cell regulation.

**Figure 3:**
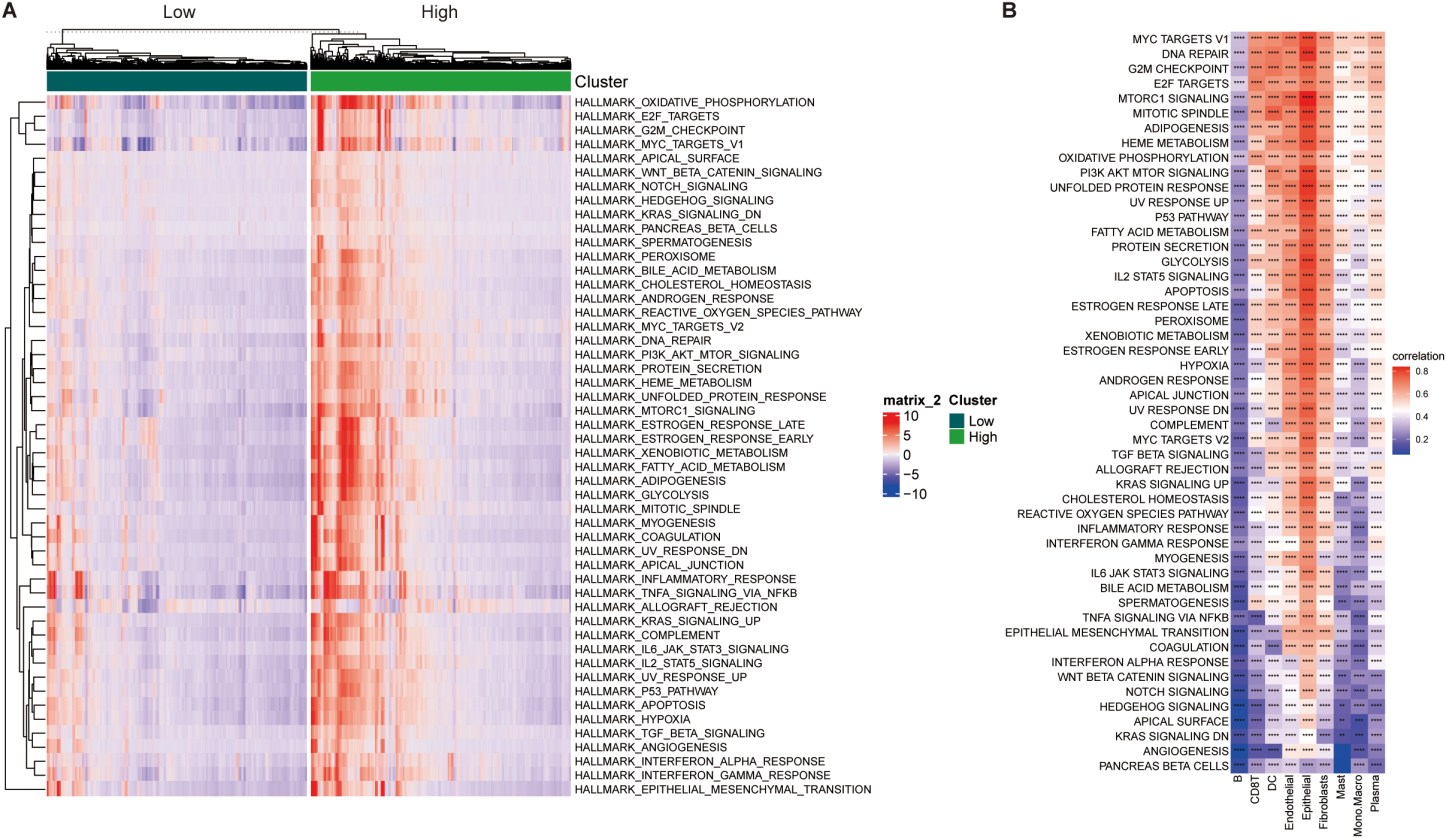
Correlation analysis between SUMOylation activity scores and hallmark pathway scores. **(A)** Heatmap displaying hallmark pathway scores for high and low SUMOylation activity score groups. Scores were derived from 50 hallmark pathways in the MSigDB database. **(B)** Heatmap illustrating the correlation between SUMOylation activity scores and hallmark pathway scores across individual cell clusters **p<0.01, ***p<0.001, ****p<0.0001.

Functionally, pathway enrichment analysis revealed that high SUMOylation activity correlated with enhanced signaling in proliferation, metabolism, and cell cycle-related pathways, particularly the G2M_CHECKPOINT, E2F_TARGETS, and MYC_TARGETS V1/V2 pathways (**Figure 3A**), suggesting that SRGs may facilitate tumor progression by promoting cell growth and metabolic activity.

Correlation analyses further linked SRG scores positively with oncogenic pathways such as MYC targets, DNA repair, and mTORC1 signaling, predominantly in epithelial cells, while B cells exhibited an inverse relationship with SUMOylation (**Figure 3B**). Together, these findings highlight the context-dependent influence of SUMOylation in modulating tumor cell behavior and shaping the immune microenvironment, particularly through its impact on B cell dynamics.

### 3.3 SRGs-based molecular classification reveals prognostic and immunological heterogeneity in STAD

**Figure 4:**
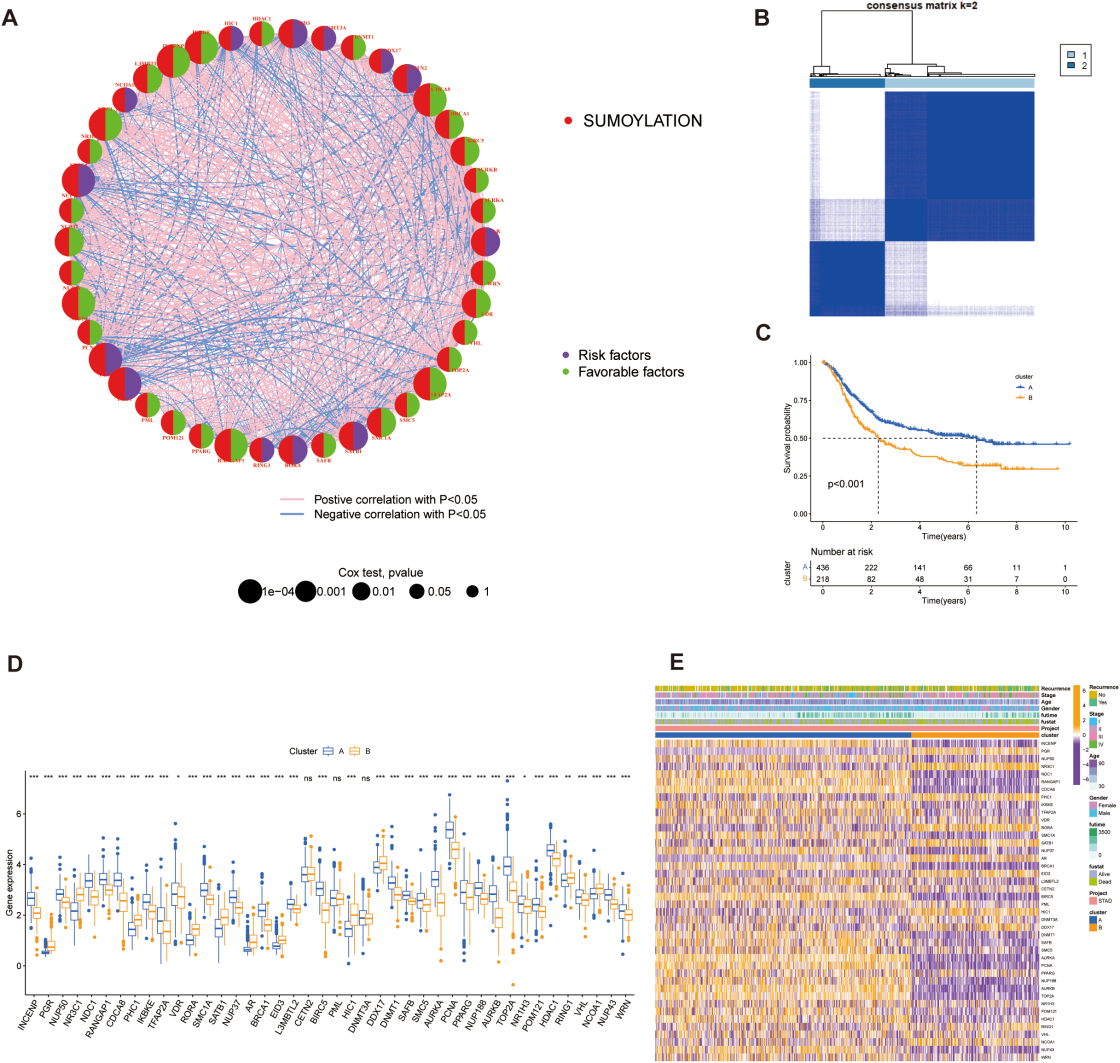
Identification of SUMOylation-related genes (SRGs) and their prognostic significance. **(A)** Correlation and univariate regression analyses of 42 SRGs (Cox *p* values < 0.001). Purple nodes indicate prognostic risk factors, whereas green nodes represent protective factors. Connecting lines between nodes denote correlations among the genes. **(B)** Unsupervised clustering analysis categorizes the 42 genes into two distinct subtypes. **(C)** Kaplan-Meier survival analysis comparing the two subtypes, highlighting differences in overall survival. **(D)** Differential gene expression analysis between the two subtypes, illustrating significant transcriptional variations. **(E)** Heatmap depicting the association between gene expression patterns and the identified subtypes, with hierarchical clustering applied to both genes and samples. *p<0.05, **p<0.01, ***p<0.001, ns, not significant.

Based on TCGA-STAD and GSE62254 datasets, we curated a total of 179 SRGs for integrated analysis. Through univariate Cox regression analysis (*p* < 0.001), 42 SRGs were identified as significantly prognosis-related (*p* < 0.001), including 13 risk and 29 favorable genes, and their functional interaction analysis revealed strong connectivity (**Figure 4A**; **Supplementary Figure 1A**).

These SRGs were subjected to unsupervised consensus clustering, which revealed two robust molecular subtypes, designated as Group A and Group B (**Figure 4B**; **Supplementary Figure 1B**). Kaplan–Meier survival analysis revealed that patients in Cluster A exhibited significantly better overall survival than those in Cluster B (*p* < 0.001, **Figure 4C**). Stratified survival analysis further confirmed that high expression of favorable SRGs was associated with improved prognosis (**Supplementary Figure 1C**; *p* < 0.001). These results suggest that the prognostic value of SRGs as potential biomarkers.

Expression comparisons between groups showed clear subtype-specific patterns: Group B exhibited elevated expression of risk factors and lower favorable genes in Group A (**Figure 4D**). Three genes (CETN2, PML, and DNMT3A) did not display significant differential expression. Clinical parameters such as recurrence status, tumor stage, age, gender, and survival outcomes were also differentially distributed, reinforcing the clinical relevance of this molecular classification (**Figure 4E**).

**Figure 5:**
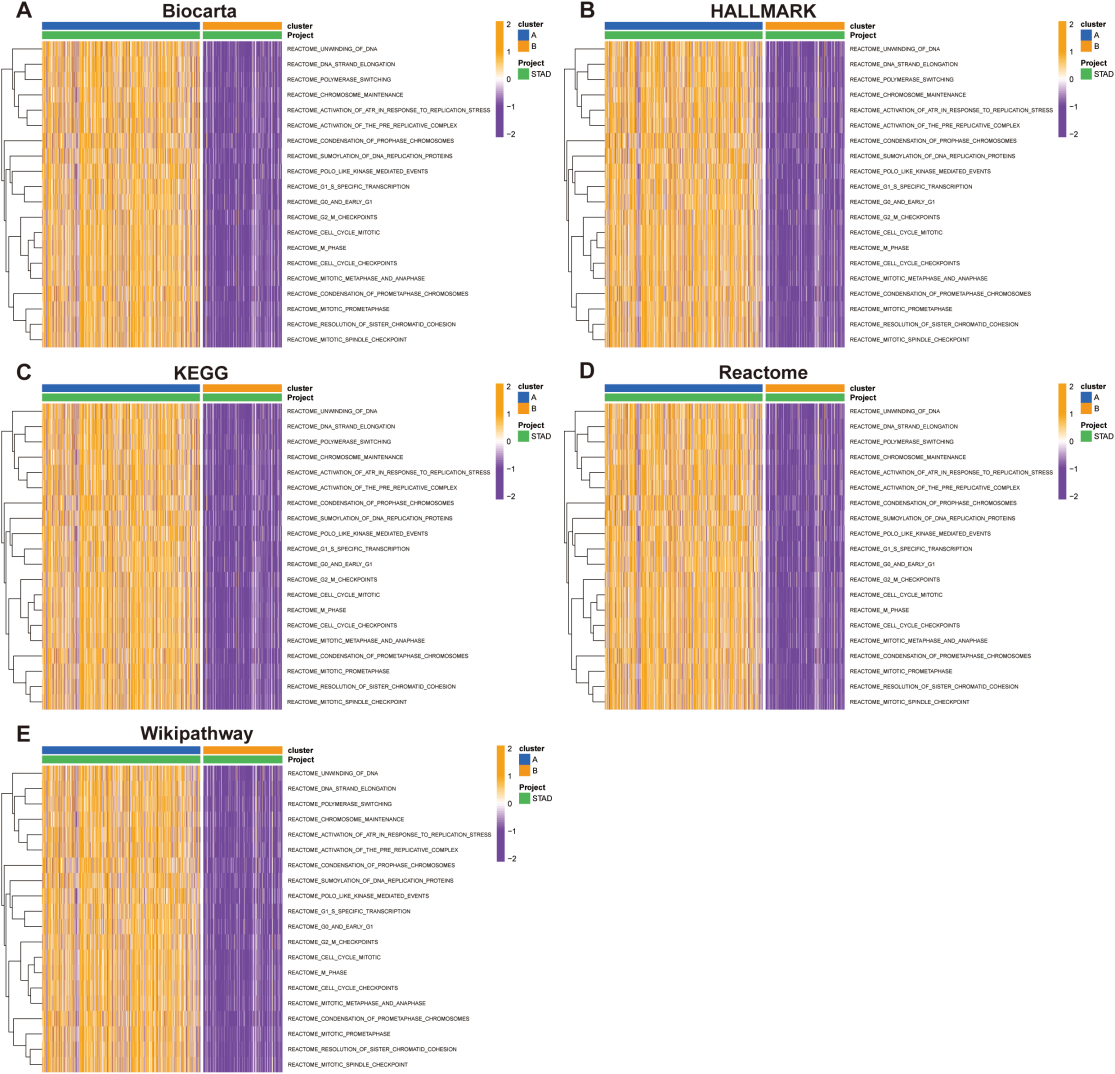
Pathway enrichment analysis of Group A and Group B across multiple databases. Pathway activity was evaluated using the Gene Set Variation Analysis (GSVA) tool in R. **(A)** Biocarta, **(B)** HALLMARK, **(C)** KEGG, **(D)** Reactome, and **(E)** WikiPathways.

To elucidate the functional differences between the subtypes, we performed GSVA using multiple pathway databases, including Biocarta, HALLMARK, KEGG, Reactome, and WikiPathways (**Figures 5A–E**). Cluster B was enriched in cell proliferation and oncogenic signaling pathways, such as E2F targets, G2M checkpoint, and MYC signaling. These findings indicate that SUMOylation-based molecular subtypes are strongly associated with both prognosis and distinct biological behaviors in STAD.

### 3.4 Distinct immune landscapes between SRGs-based STAD subtypes

**Figure 6:**
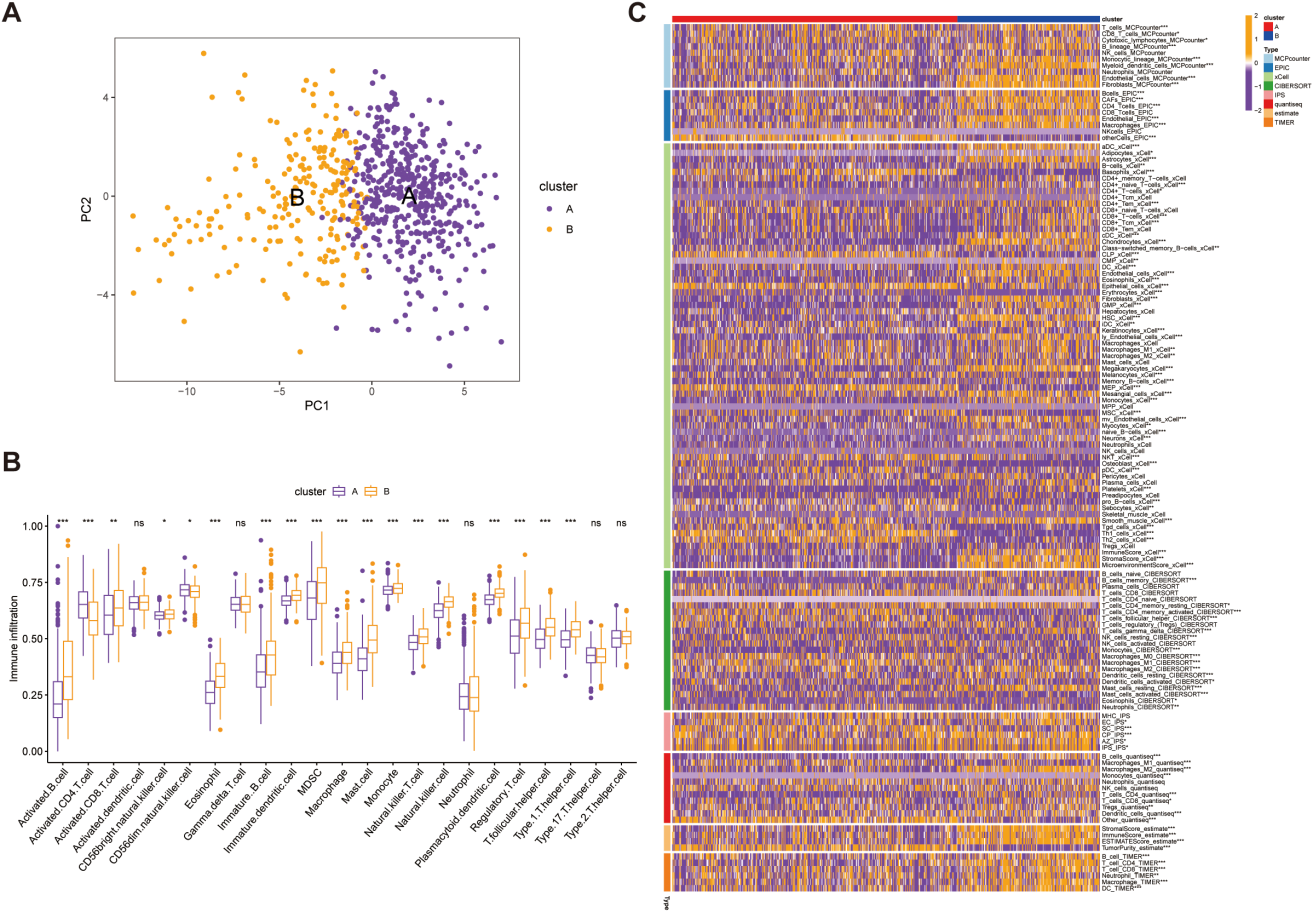
Validation of immune microenvironment differences between Group A (450 samples) and Group B (225 samples). **(A)** Principal Component Analysis (PCA) plot illustrating the distinct separation between Group A and Group B based on immune microenvironment profiles. **(B)** Comparative analysis of immune cell infiltration levels between Group A and Group B, highlighting significance differences in immune cell composition. **(C)** Heatmap depicting the association between the identified subtypes and immune cells in cell populations within the immune microenvironment, with hierarchical clustering applied to visualize patterns. *p<0.05, **p<0.01,***p<0.001, ns, not significant.

Our PCA-driven dimensionality reduction analysis further validated the robustness of our classification, yielding distinct clustering patterns for Group A (450 samples) and Group B (225 samples) from the merged data, thereby confirming the accuracy of our categorization approach (**Figure 6A**). To explore differences in the tumor immune microenvironment (TIME) between the two subtypes, we quantified immune cell infiltration using the single-sample gene set enrichment analysis (ssGSEA) method. We observed significant differences in the infiltration levels of 18 immune cell types between the two groups (**Figure 6B**). Additionally, Group B exhibited elevated infiltration in the majority of immune cell populations, except for activated CD4+ T cells and CD56^dim^ natural killer (NK) cells, which were more abundant in Group A. Furthermore, integration of eight established immune cell estimation algorithms demonstrated consistent divergence in immune composition between the subtypes (**Figure 6C**). Together, these findings indicate that SUMOylation is a key regulator of the tumor microenvironment, particularly influencing immune dynamics and tumor progression.

### 3.5 SUMOylation risk score accurately predicts prognosis in STAD

**Figure 7:**
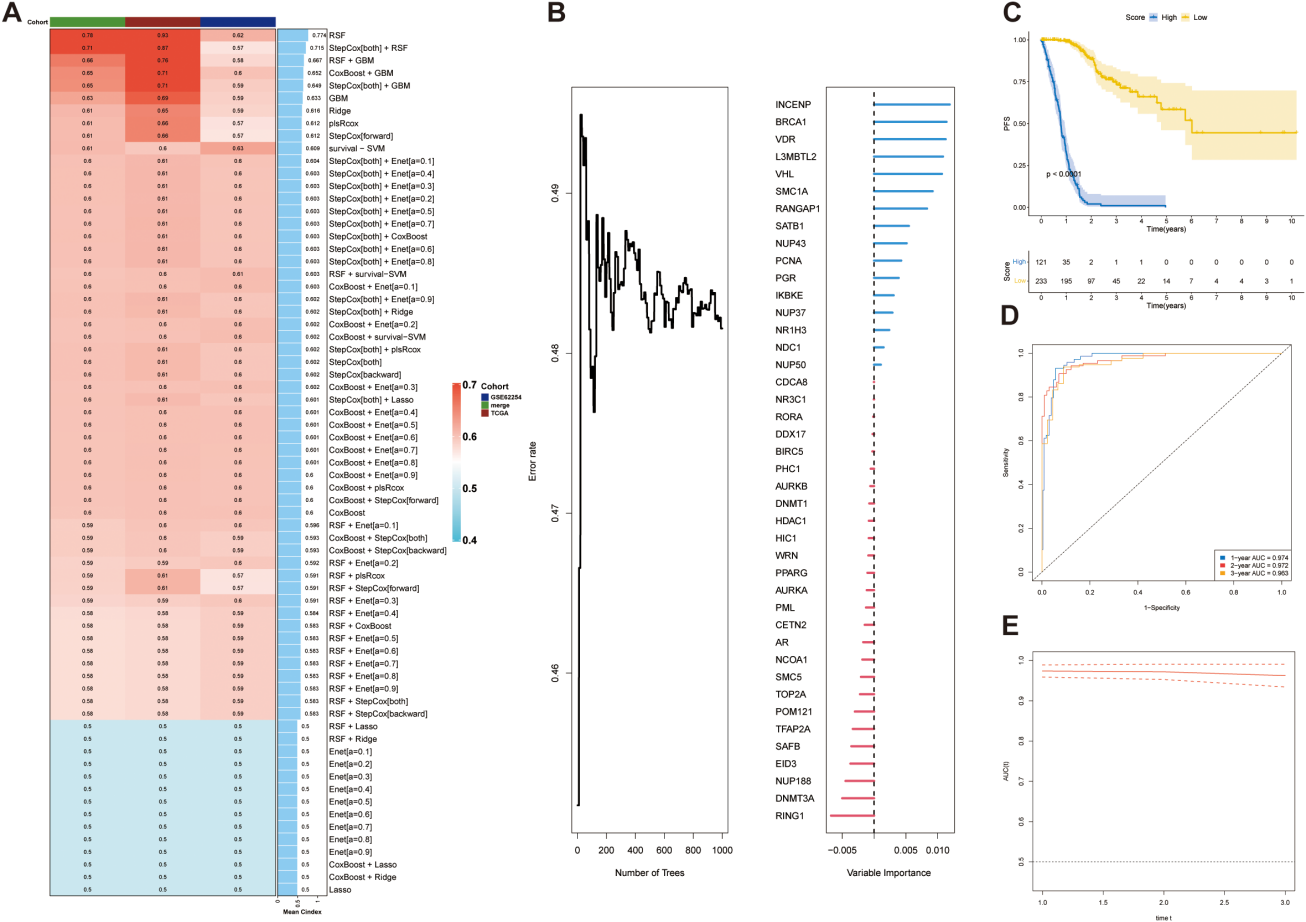
Construction and validation of a prognostic model based on SUMOylation-related genes. **(A)** Concordance index (C-index) analysis demonstrating the performance of the prognostic model. The TCGA dataset was used as the training set, while external datasets were used for validation. **(B)** Ket genes identified by random forest analysis and their contribution to the prognostic scoring model. **(C)** Prognostic risk stratification analysis in the training cohort (TCGA dataset). **(D)** Kaplan–Meier survival curve for the training set, stratified by high-risk and low-risk groups. **(E)** Time-dependent receiver operating characteristic (ROC) curve for the training set, evaluating the model’s predictive accuracy at 1, 2, and 3 years.

Based on the 42 prognosis-associated SUMOylation-related genes (SRGs) identified in the earlier analysis (**Figure 4**), we constructed a SUMOylation Risk Score (SRS) model to predict patient outcomes. To ensure robustness and generalizability, a total of 69 machine learning models were developed using 10 algorithmic frameworks. Among these, the random survival forest (RSF) model achieved the best performance, with the highest average C-index of 0.774 (**Figure 7A**). We ranked these genes according to their variable importance derived from the RSF model, and illustrated the expression patterns of the top 10 key genes at the single-cell level resolution (**Figure 7B**; **Supplementary Figure 2**). Using the median SRS value as a cutoff, patients were stratified into high- and low-risk groups. Survival analysis showed significantly worse outcomes in the high-risk group (*p <*0.0001) (**Figure 7C**). The RSF-based model demonstrated excellent predictive power, with AUCs of 0.974 and 0.963 for one- and three-year survival, respectively (**Figures 7D, E**). These findings were consistently validated in both the independent validation cohort and the combined dataset (**Supplementary Figures 3A, B**). Together, these findings confirm that the SRS model derived from SRGs provides a reliable and accurate tool for survival prediction in STAD, with potential utility in risk stratification and individualized treatment planning.

### 3.6 SRS prognostic model correlates with clinical outcomes and tumor progression

**Figure 8:**
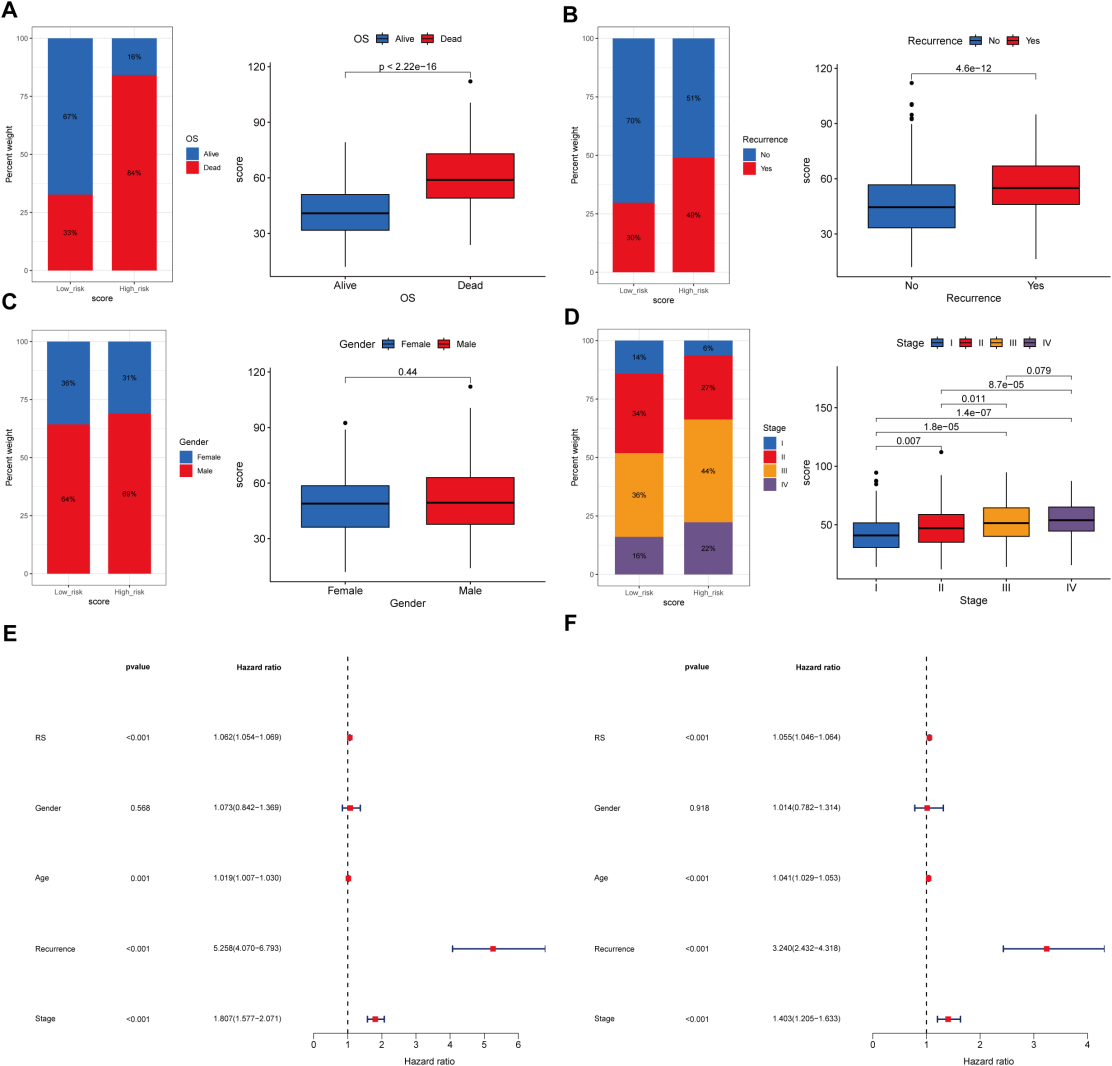
Association between the prognostic model score and clinical characteristics. **(A)**Kaplan-Meier survival curves stratified by the prognostic model score, illustrating the relationship between model score and overall survival (OS). **(B)** Distribution of the model score stratified by recurrence. **(C)** Distribution of the model score stratified by gender. **(D)** Distribution of the model score stratified by tumor stage. **(E, F)** Univariate and multivariate regression analyses demonstrating the prognostic model score as an independent predictor of clinical outcomes.

Analyses revealed distinct distributions of model scores across clinical features, including survival status (Alive vs. Dead), recurrence status (No vs. Yes), sex (Female vs. Male), and tumor stage (Stage I-IV) (**Figures 8A–D**). The high-risk group exhibited a significantly higher proportion of deceased patients compared to the low-risk group (Alive: 16% vs. 67%; Dead: 84% vs. 33%; *p* < 0.001), along with a higher recurrence rate (Recurrence: 49% vs. 30%; *p* < 0.001). Advanced tumor stages were more prevalent in the high-risk group, with Stage IV representing 22% versus 16% in the low-risk group (Stage IV: 22% vs. 16%; *p* < 0.001). However, no significant gender-based difference in risk scores was observed (*p* = 0.44). These findings established SRGs as independent prognostic indicators for overall survival (OS).

Furthermore, both univariate and multivariate Cox regression analyses confirmed the significant association between model-derived risk scores (RS) and OS (HR = 1.062, 95% CI: 1.054–1.069, *p* < 0.001; HR = 1.055, 95% CI: 1.046–1.064, *p* < 0.001, respectively) (**Figures 8E, F**). These findings collectively suggest that the SRGs-based model RS maintains significantly correlation with STAD progression and serves as an independent prognostic indicator for patient outcomes.

### 3.7 Low-risk group exhibits enrichment in genome stability and cell cycle regulation pathways

**Figure 9:**
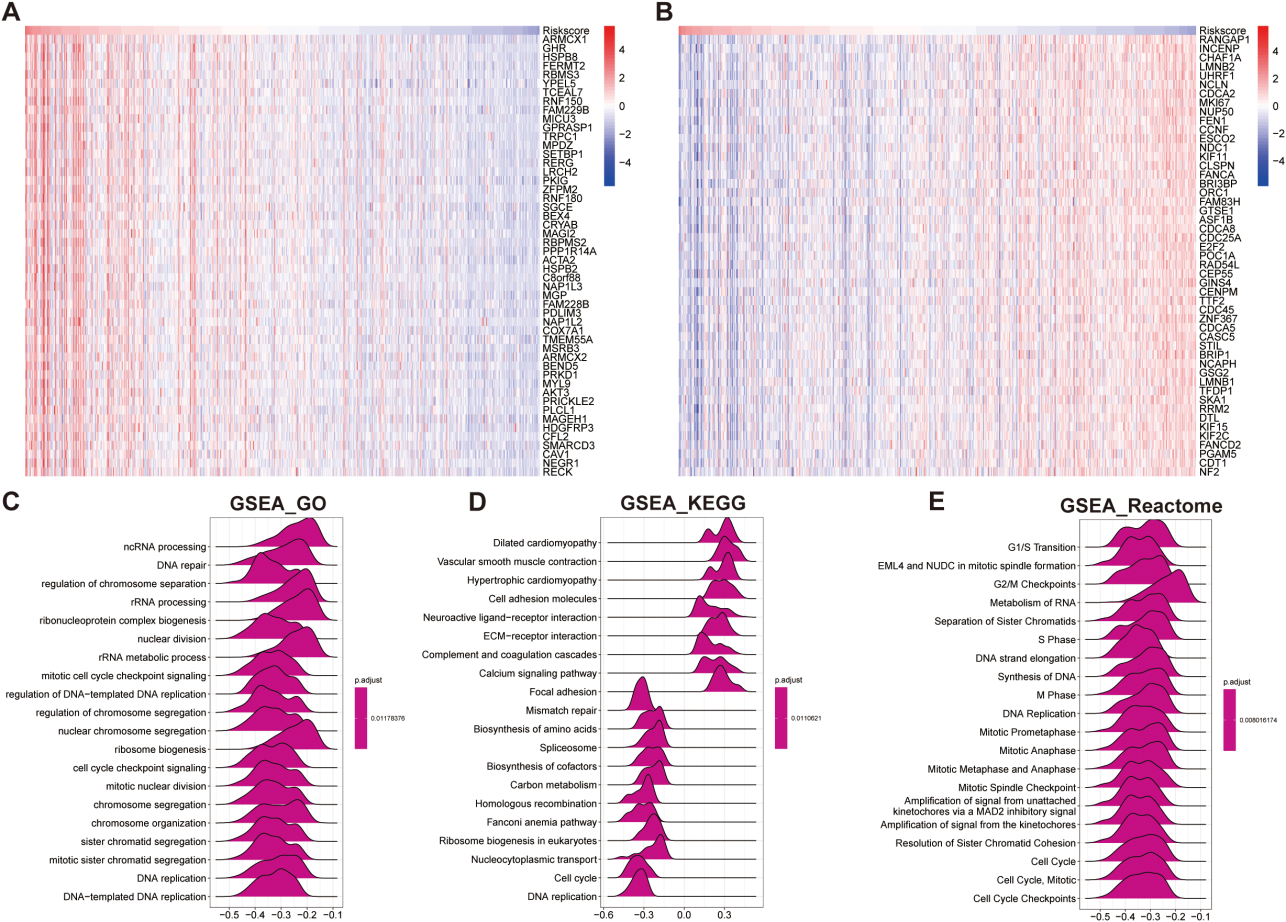
Functional enrichment analysis based on the prognostic model score. **(A, B)** Heatmaps showing the top 50 positively correlated genes **(A)** and negatively correlated genes **(B)** with the prognostic model score. **(C–E)** GSEA based on the correlation analysis results, highlighting significantly enriched pathways in: **(C)** GO terms, **(D)** KEGG pathways, and **(E)** Reactome pathways.

Biological pathway analyses stratified by risk scores revealed distinct molecular signatures between groups (**Figures 9A, B**). Gene Set Enrichment Analysis (GSEA) showed that the low-risk group was significantly enriched in biological processes related to genome stability and cell cycle regulation (**Figures 9C–E**). GO analysis revealed significant enrichment in genome stability-related pathways, such as DNA repair, nuclear division, and chromosome segregation in the low-risk group (**Figure 9C**). KEGG analysis further confirmed that the low-risk group was enriched in pathways crucial for DNA damage repair and cell cycle regulation, including mismatch repair, homologous recombination, Fanconi anemia pathway, and DNA replication (**Figure 9D**). Reactome pathway analysis corroborated these findings, showing a predominance of cell cycle control and chromosomal maintenance pathways in the low-risk group (**Figure 9E**).

Overall, these results suggest that STAD patients in the low-risk group maintain more stable genomic integrity and effective cell cycle regulation, which may contribute to a lower malignant potential and better prognosis (**Figure 9**).

### 3.8 High-risk group displays a dysfunctional and immune-excluded tumor microenvironment

**Figure 10:**
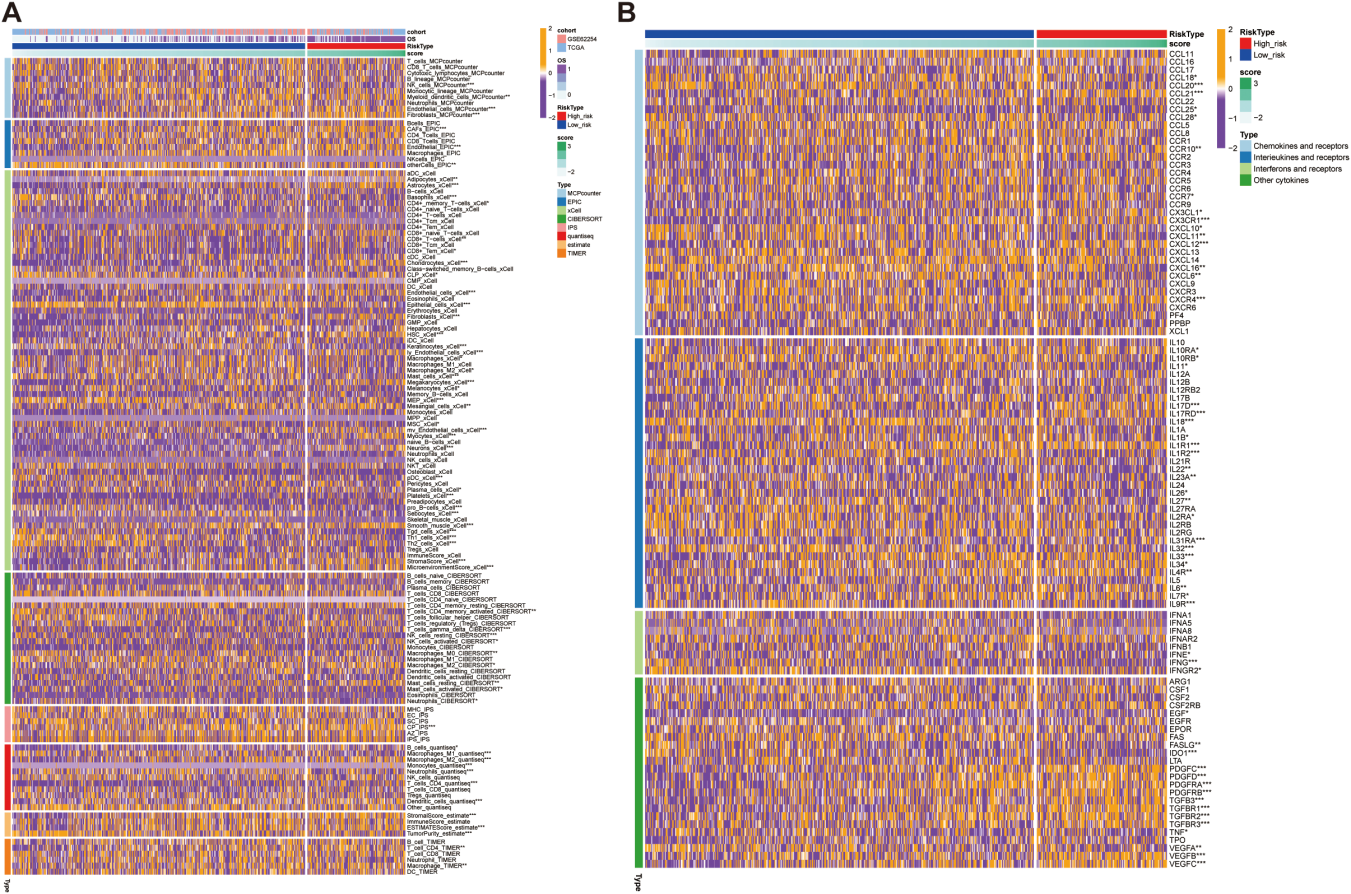
Immune infiltration and cytokine expression profiles in high- and low- risk groups. **(A)** Heatmap showing the correlation between the prognostic model score and immune cells infiltration levels, assessed using eight computational methodologies. **(B)** Heatmap showing the correlation between the prognostic model score and the expression of immune-related molecules, including chemokines and their receptors. High-score groups exhibit low immune infiltration, predicting poor response to immunotherapy. *p<0.05, **p<0.01, ***p<0.001.

To gain further insight into the tumor immune microenvironment, we implemented eight computational methodologies (MCPcounter, EPIC, xCell, CIBERSORT, IPS, quanTIseq, ESTIMATE, and TIMER) to assess the tumor infiltration. The associations between model-derived risk scores and immune cell populations were systematically visualized through comprehensive heatmap analysis (**Figure 10A**). Employing the CIBERSORT algorithm, CD4+ T cells and macrophages were significantly reduced in the high-risk group. Additionally, ESTIMATE results showed reduced tumor purity but elevated immune and stromal scores in the high-risk group, indicating a more complex and heterogeneous immune landscape (**Figure 10A**). Interestingly, although certain immune cell types were increased in the high-risk group, the overall level of immune cell infiltration was lower than that in the low-risk group, potentially reflecting reduced sensitivity to immunotherapy in this subgroup (**Figure 10A**). To further delineate immune-related differences, we compared the expression profiles of various immunoregulatory molecules, including chemokines and their receptors, interleukins, interferons, and other cytokine families, between the two risk groups (**Figure 10B**). This comprehensive evaluation provided additional insights into the relationship between the immune microenvironment characteristic and the identified risk groups (**Figure 10B**). These findings collectively suggest that the high-risk group may exhibit features of immune exclusion or dysfunction, which could compromise the effectiveness of immune checkpoint blockade.

### 3.9 High-risk group exhibits immune evasion and reduced sensitivity to anti-tumor therapies

**Figure 11:**
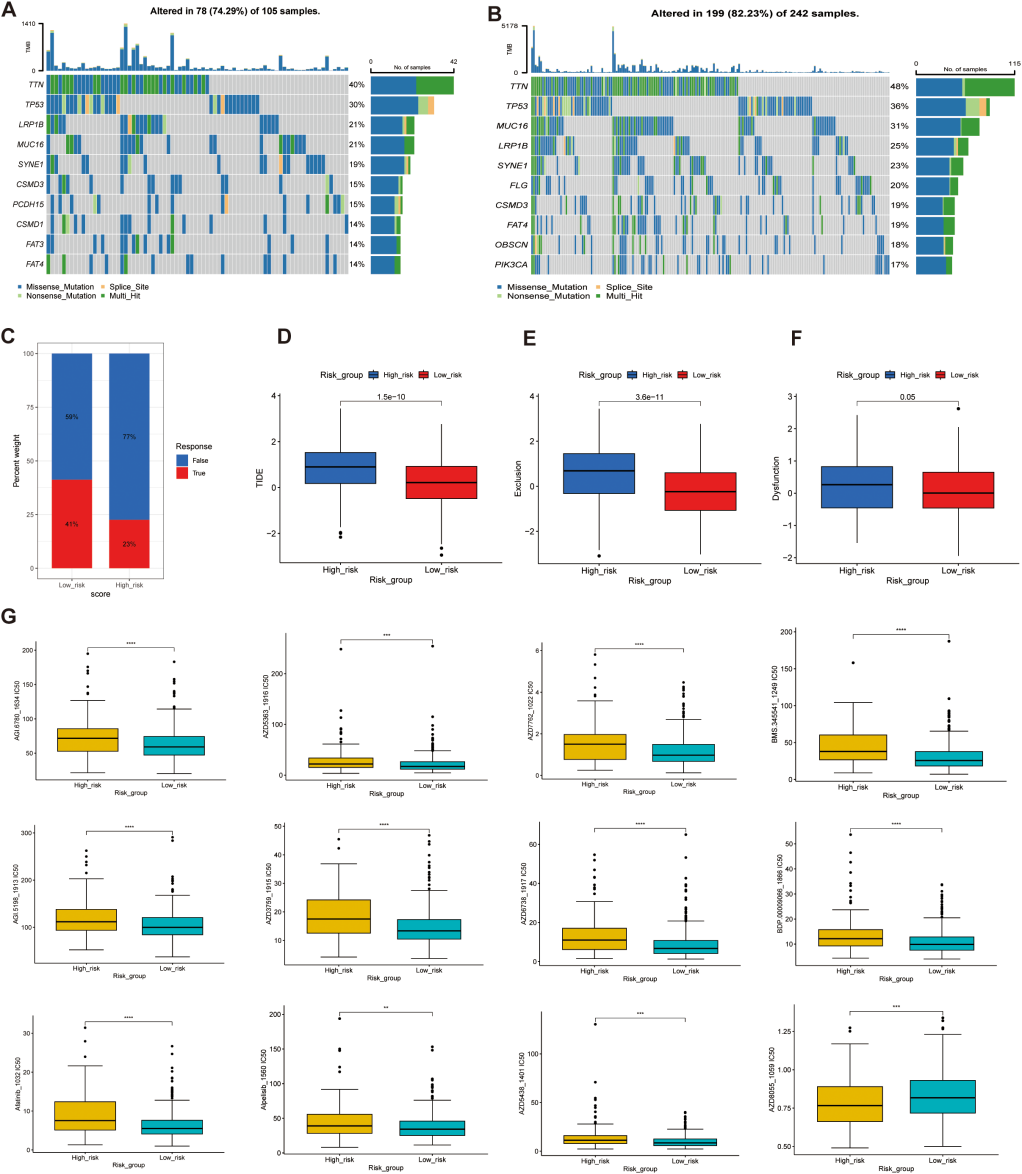
Genetic alterations, immunotherapy response, and drug sensitivity analysis in high- and low-risk groups. **(A, B)** Mutation landscape analysis in the high-risk **(A)** and low-risk **(B)** groups, highlighting the mutated genes and their mutation frequencies. **(C–F)** Tumor Immune Dysfunction and Exclusion (TIDE) analysis, including: **(C)** TIDE scores, **(D)** Exclusion scores, **(E)** Dysfunction scores, and **(F)** TME analysis. **(G)** Drug sensitivity analysis, comparing the half-maximal inhibitory concentration (IC50) values of various anticancer drugs between the high-score and low-score groups. Higher IC50 values indicate lower sensitivity **p<0.01, ***p<0.001, ****p<0.0001.

To investigate the differences in immunotherapy response and drug sensitivity between the high- and low-risk groups, we performed somatic mutation analysis using the “MAFTOOL” R package. TTN, TP53, LRP18, MUC16, SYNE1 were identified as the top three genes with the highest mutation frequencies in both risk groups (**Figures 11A, B**). Odds ratio (OR) analysis supported this finding, indicating a significantly lower mutation probability in the low-risk group (**Supplementary Figure 4A**). With respect to tumor biological features, mRNAsi scores decreased as risk scores increased, suggesting that that the high-risk group exhibits reduced cellular stemness. No significant difference in microsatellite instability (MSI) was observed between the groups. However, the tumor mutational burden (TMB) was significantly lower in the high-risk group compared to the low-risk group, potentially indicating poorer immunotherapy responsiveness (**Supplementary Figures 4B–D**). In evaluating immunotherapy response indicators with four analyses, 41% of the low-risk samples were predicted to respond to immune checkpoint blockade, compared to only 23% of the high-risk samples (**Figure 11C**). Moreover, the high-risk group exhibited a significantly higher immune exclusion score and a slightly elevated immune dysfunction score, alongside a markedly higher TIDE score (*p* < 0.001), reflecting an immune microenvironment prone to exclusion and dysfunction (**Figures 11C–F**). These features collectively suggest a greater likelihood of immune evasion and impaired immunotherapeutic efficacy in high-risk patients. Finally, analysis of drug response sensitivity revealed that the high-risk group had significantly higher estimated IC50 values for several antitumor agents (e.g., BMS-345541, BX-912, and AZD7762), indicating reduced drug sensitivity. Conversely, the low-risk group exhibited higher IC50 values for agents such as AZD8055 (**Figure 11G**). Overall, these findings highlight substantial differences in mutation landscapes, immune evasion potential, and drug responsiveness between the two groups, suggesting that patients in the high-risk group may experience greater immune suppression and diminished sensitivity to both immunotherapy and certain chemotherapeutic agents.

### 3.10 Knockdown of L3MBTL2 or VHL enhances proliferation and invasion in STAD cells

**Figure 12:**
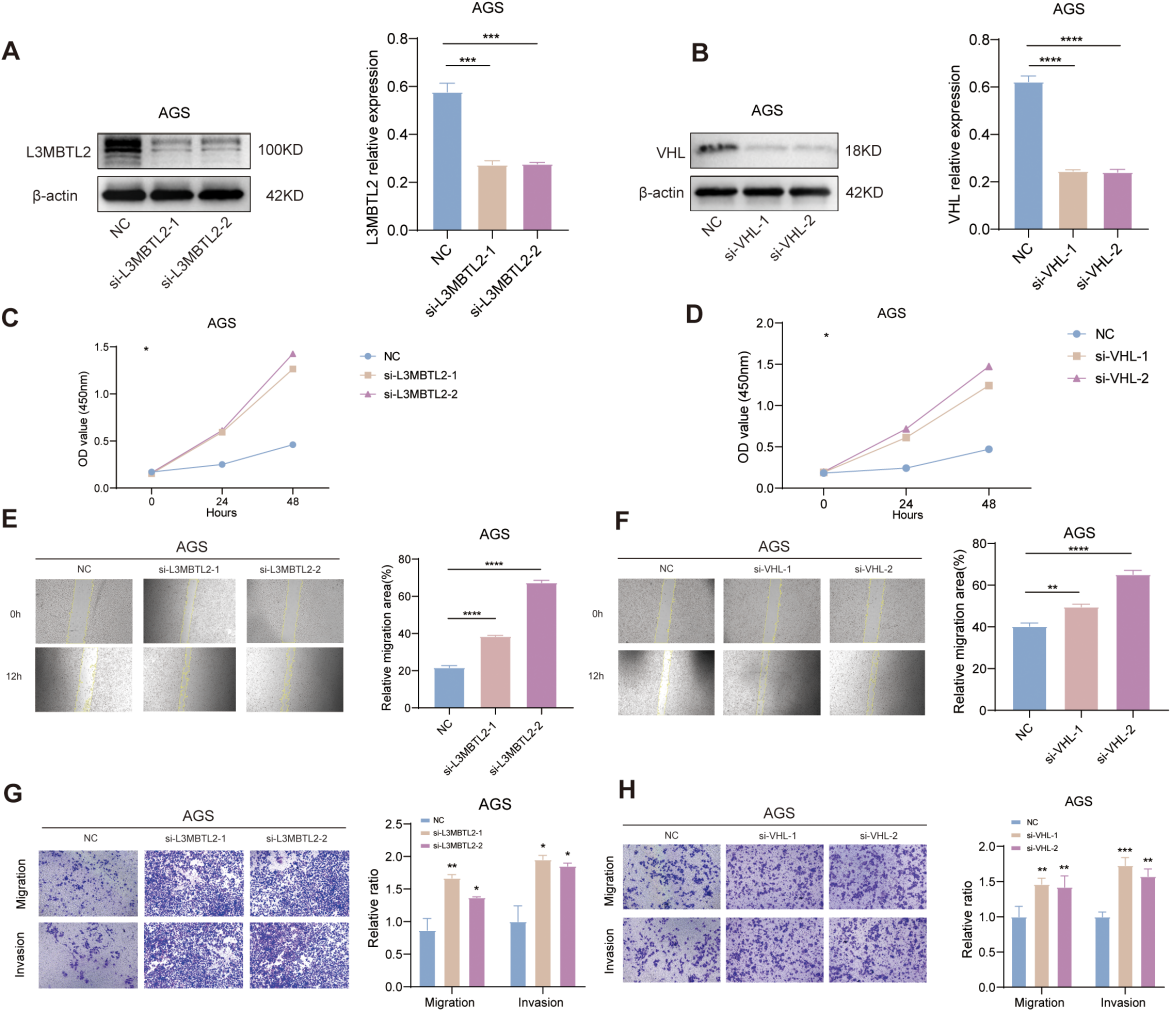
Functional validation of L3MBTL2 and VHL as tumor suppressors in STAD. **(A, B)** Western blot analysis confirming efficient knockdown of L3MBTL2 **(A)** and VHL **(B)** protein levels in AGS cells transfected with shRNA plasmids via PEI-mediated transfection. β-actin served as a loading control. **(C, D)** CCK-8 proliferation assays showing enhanced cell viability in L3MBTL2- **(C)** and VHL-knockdown **(D)** cells compared to scramble controls at 0, 24 and 48 hours (*p* < 0.01, two-way ANOVA). **(E, F)** Wound healing assays demonstrating accelerated migration in L3MBTL2- **(E)** and VHL-depleted **(F)** cells at 12 hours post-scratch. Quantified wound closure rates are shown (mean ± SD; *p* < 0.01, unpaired t-test). **(G, H)** Transwell migration (upper panels) and Matrigel-based invasion (lower panels) assays revealing increased migratory and invasive capacities of L3MBTL2- **(G)** and VHL-knockdown **(H)** cells. Representative images (left) and quantifications (right; mean ± SD; *p* < 0.01, unpaired t-test) are shown. All experiments were performed in triplicate. NC: negative control. *p<0.05, **p<0.01, ***p<0.001, ****p<0.0001.

To date, the functional roles of L3MBTL2 and VHL in the pathogenesis of STAD remain largely unexplored. In our research, AGS cells with transient knockdown of L3MBTL2 or VHL exhibited significantly reduced protein levels, as confirmed by Western blotting (**Figures 12A, B**). Functional assays revealed that knockout of L3MBTL2 and VHL significantly enhanced cell proliferation, as indicated by increased absorbance in the CCK-8 assays (**Figures 12C, D**). Furthermore, wound healing assays demonstrated accelerated wound closure in L3MBTL2- or VHL-knockdown cells, indicating enhanced migratory capacity (**Figures 12E, F**). Consistently, transwell migration and invasion assays showed that silencing L3MBTL2 or VHL significantly promoted AGS cell migration and invasion (**Figures 12G, H**).

These findings collectively suggest that L3MBTL2 and VHL may function as tumor suppressors in STAD by restraining cell proliferation, migration, and invasion. Their loss may contribute to malignant progression and thus represent potential therapeutic targets in STAD.

## 4 Discussion

Stomach adenocarcinoma (STAD) is a globally prevalent malignancy with high mortality, and its early detection remains challenging due to non-specific or absent clinical symptoms (39–41),. Thus, identifying robust biomarkers and novel molecular subtypes is essential for improving patient outcomes. SUMOylation, a dynamic post-translational modification (PTM), plays multifaceted roles in cancer biological processes (10, 42, 43). However, its specific role in STAD has not been comprehensively explored.

To address this, we applied an integrative approach combining single-cell RNA sequencing (scRNA-seq) with bulk transcriptomic data to characterize SUMOylation-related genes (SRGs) and their biological significance in STAD. SRGs are predominantly expressed in epithelial tumor cells, driving key oncogenic pathways such as epithelial-mesenchymal transition (EMT), KRAS signaling, and IL6-JAK-STAT3 activation, highlighting their contribution to tumor progression and invasiveness. Elevated SUMOylation levels were negatively correlated with B cell infiltration and TNFα/NF-κB signaling, indicating a potential contribution to immune evasion. Notably, epithelial cells with high SUMOylation activity exhibited hyperactivation of proliferative pathways (e.g., E2F_TARGETS, G2M_CHECKPOINT), further supporting the role of SUMOylation as a metabolic regulator promoting tumor aggressiveness. These findings align with prior studies linking SUMOylation to metastasis and chemoresistance in gastrointestinal cancers (11, 44, 45). Based on the expression of 42 SRGs, we identified two molecular subtypes of STAD. Cluster B tumors displayed activated oncogenic pathways and impaired DNA repair mechanisms, correlating with significantly worse prognosis.

To further quantify the clinical utility of SRGs, we constructed a SUMOylation Risk Score (SRS) model using a comprehensive machine learning framework, incorporating 10 algorithms and 69 combination strategies. This robust model identified 42 prognostically relevant genes, including 16 with high feature importance, such as INCENP, BRCA1, VDR, L3MBTL2, and VHL.

Many of these genes are mechanistically linked to SUMOylation-related processes. For instance, INCENP activates Aurora B and promotes its SUMOylation (46, 47). BRCA1, acts as a SUMO E3 ligase involved in DNA damage response in gastric cancer (48, 49). VDR is SUMOylated by SENP1 and SENP2, while L3MBTL2 stabilizes RBPJ binding to Notch genes via the SUMO-modified PRC1.6 complex (50, 51). RANGAP1 is SUMOylated with SUMO-1, enhancing its interaction with RanBP2 and regulates its role in the Ran GTPase cycle (52). SATB1 is SUMOylated at lysine-744, a modification regulated by PIAS1, which controls its cleavage by caspase-6 at PML nuclear bodies (53). PCNA, when modified by SUMO, exhibits increased conformational flexibility, facilitating the recognition of effector proteins and the formation of PCNA tool belts (54). PGR, as a member of the steroid hormone receptor (SHR) family, undergoes SUMOylation and phosphorylation (S294, S400), which regulate its interaction with growth factor signaling, subcellular localization, and degradation (55, 56). Additionally, ubiquitination at the K388 site inhibits ERα activity, IKBKE is involved in NF-κB activation through NEMO SUMOylation (57). NDC1 and NUP50 play roles in SUMO-regulated DNA repair, with NUP50 affecting nonhomologous end joining but not 53BP1 sumoylation (58, 59). SMC1A accelerates gastric cancer (GC) progression by upregulating SNAIL, thereby promoting EMT and enhancing cell proliferation, migration, and invasion (60). In GC, NUP43 is closely linked to prognosis, especially in the high-risk group, where its expression correlated with immune scores, immune cell infiltration, and the enrichment of cancer and immune pathways (61). NUP37 enhances GC cell proliferation, migration, and invasion by activating the PI3K/AKT/mTOR signaling pathway (62). Taken together, these functional associations reinforce the biological plausibility of our model and underscore the central role of SUMOylation in STAD tumorigenesis.

In terms of mutational landscapes, TTN, TP53, LRP1B, MUC16, and SYNE1 were the most frequently mutated genes in both risk groups, with higher mutation frequencies observed in the low-risk group. TTN, TP53, LRP1B, and SYNE1 are frequently mutated not only in solid tumors but also in hematologic malignancies (63–71). MUC16 promotes ovarian cancer progression by inducing an inflammatory and immunosuppressive neutrophil phenotype and playing a critical role in immune modulation and disease prediction (72–74).

Beyond prognostication, the SRS model demonstrated clinical relevance by correlating with tumor stage, recurrence risk, and response to therapy. High-SRS patients exhibited advanced-stage disease, worse overall survival, and poor response to immune checkpoint inhibitors (ICIs).

These differences were further reflected in somatic mutation profiles and tumor mutational burden (TMB), with the high-risk group showing a lower TMB and fewer mutations. Consistently, the high-SRS group also exhibited elevated immune dysfunction and exclusion scores, features associated with reduced sensitivity to immunotherapy. Drug sensitivity analysis revealed potential resistance of the high-risk group to several chemotherapeutic and targeted agents, suggesting that personalized or alternative therapeutic strategies may be required for these patients.

To experimentally validate the biological relevance of key SRGs, we selected two representative genes, L3MBTL2 and VHL, for functional assays based on their strong prognostic value and limited prior characterization in STAD. Knockdown of either gene significantly enhanced proliferation, migration, and invasion *in vitro*. These findings are consistent with previous reports of L3MBTL2 acting as a metastasis suppressor through chromatin remodeling (75–77), as well as VHL functioning as a negative regulator of angiogenesis via HIF-1α degradation (78, 79) Our results therefore provide *in vitro* evidence supporting their tumor-suppressive roles in STAD.

Interestingly, while epidemiological studies have shown a male predominance in gastric cancer incidence (80, 81), our analysis revealed no significant sex-related differences in SRS distribution, suggesting that SUMOylation-related transcriptional programs may operate independently of sex hormone signaling. Further studies focusing on hormone receptor expression or subtype-specific effects may help clarify this observation.

In summary, our study presents a comprehensive characterization of the SUMOylation landscape in STAD and highlights the translational potential of SRGs. By integrating scRNA-seq with bulk RNA-seq, we developed a biologically grounded and clinically applicable SRS model that stratifies patients by prognosis and therapeutic responsiveness. However, several limitations must be acknowledged: (1) our functional validation was limited to AGS cells and lacked *in vivo* verification; (2) spatial and temporal dynamics of SUMOylation in the tumor immune microenvironment were not addressed. Future research incorporating organoid systems, animal models, and spatial transcriptomics will be essential for fully elucidating the therapeutic potential of SUMOylation in STAD.

## 5 Conclusions

This study highlights the pivotal role of SUMOylation-related genes (SRGs) in the progression and immune landscape of stomach adenocarcinoma (STAD). By integrating single-cell and bulk transcriptomic data, we developed a robust SUMOylation risk score (SRS) model that effectively predicts prognosis and treatment response. Functional validation of key SRGs, including L3MBTL2 and VHL, confirmed their tumor-suppressive roles. These findings provide new insights into SUMOylation as a potential biomarker and therapeutic target in STAD.

## Data Availability

The original contributions presented in the study are included in the article/**Supplementary Material**. Further inquiries can be directed to the corresponding author/s.

## Supplementary material

Supplementary Figure 1 Univariate analysis and subtype clustering based on SUMOylation-related genes. **(A)** Forest plot showing the results of univariate Cox regression analysis for SUMOylation-related genes. **(B)** Unsupervised clustering analysis dividing samples into distinct molecular subtypes based on SUMOylation-related gene expression (k=3-6). **(C)** Representive Kaplan-Meier survival curves for favorable SUMOylation-related genes.

Supplementary Figure 2 Single-cell expression analysis of key genes identified by the random forest model. Uniform Manifold Approximation and Projection (UMAP) plot visualizing the single-cell expression patterns of the 10 key genes identified by the random forest model.

Supplementary Figure 3 Validation of the prognostic model in external cohorts. **(A)** Validation in the GSE62254 cohort: Left: Kaplan-Meier survival curves stratified by the prognostic model score. Middle: Receiver operating characteristic (ROC) curve evaluating the model’s predictive accuracy. Right: Time-dependent ROC curve assessing the model’s performance at different time points. **(B)** Validation in the merged cohort, showing consistent performance of the prognostic model.

Supplementary Figure 4 Molecular characteristics and immunotherapy response in high- and low-risk groups. **(A)** Differential gene mutation analysis between high-risk and low-risk groups, highlighting the top mutated genes and their frequencies. **(B)** Analysis of cell stemness (mRNAsi): Left: Correlation between the prognostic model score and mRNAsi values. Middle: Relationship between the prognostic model score and mRNAsi expression. Right: Distribution of mRNAsi values in high-risk and low-risk groups. **(C)** Microsatellite instability (MSI) analysis, comparing MSI levels between high-risk and low-risk groups. **(D)** Tumor mutational burden (TMB) analysis, showing lower TMB in the high-risk group, indicating a potential poor response to immunotherapy.

## Glossary

AUC: area under the curve
BP: biological process
CCK-8: Cell Counting Kit-8
CI: Confidence Interval
C-index: concordance index
COSG: cell-type orientation scoring for genes
DCs: dendritic cells
DEGs: differentially expressed genes
EMT: epithelial–mesenchymal transition
Enet: Elastic Network
FBS: Fetal Bovine Serum
FDR: false discovery rate
GBM: Gradient boosting machine
GC: gastric cancer
GEO: Gene Expression Omnibus
GO: Gene Ontology
GSVA: gene set variation analysis
*H. pylori*: *Helicobacter pylori*
IC₅₀: Half-Maximal Inhibitory Concentration
IL6: Interleukin 6
IPS: Immunophenoscore
KEGG: Kyoto Encyclopedia of Genes and Genomes
KM: Kaplan–Meier
LASSO: least absolute shrinkage and selection operator
MDSCs: myeloid-derived suppressor cells
MMR: mismatch repair
mRNAsi: mRNA stemness index
MSI: microsatellite instability
OD: optical density
OR: odds ratio
OS: overall survival
PCA: principal component analysis
PD-1: programmed death-1
PEI: polyethylenimine
PGR: progesterone receptor
PlsRcox: partial least squares regression for Cox
PR: progesterone receptor
PTM: post-translational modification
PTMs: post-translational modifications
PVDF: polyvinylidene fluoride
ROC: receiver operating characteristic
RS: risk score
RSF: random survival forest
scRNA-seq: single-cell RNA sequencing
SENPs: SUMO-special proteases
SHR: steroid hormone receptor
SRGs: SUMOylation-related genes
ssGSEA: single-sample gene set enrichment analysis
STAD: stomach adenocarcinoma
SUMOs: small ubiquitin-like modifiers
SuperPCs: Supervised Principal Components
Survival-SVM: survival support vector machine
TCGA-STAD: The Cancer Genome Atlas Stomach Adenocarcinoma
TIME: tumor immune microenvironment
TMB: tumor mutational burden
TME: tumor microenvironment
VHL: Von Hippel-Lindau
ZAP-70: Zeta-chain-associated Protein Kinase 70.
