# Supplementary figures and images for: SUMOylation-related genes define prognostic subtypes in stomach adenocarcinoma: integrating single-cell analysis and machine learning analyses
Source: Front Immunol. 2025 Aug 1;16:1527233. doi: 10.3389/fimmu.2025.1527233 (PMC12354628; doi:10.3389/fimmu.2025.1527233)

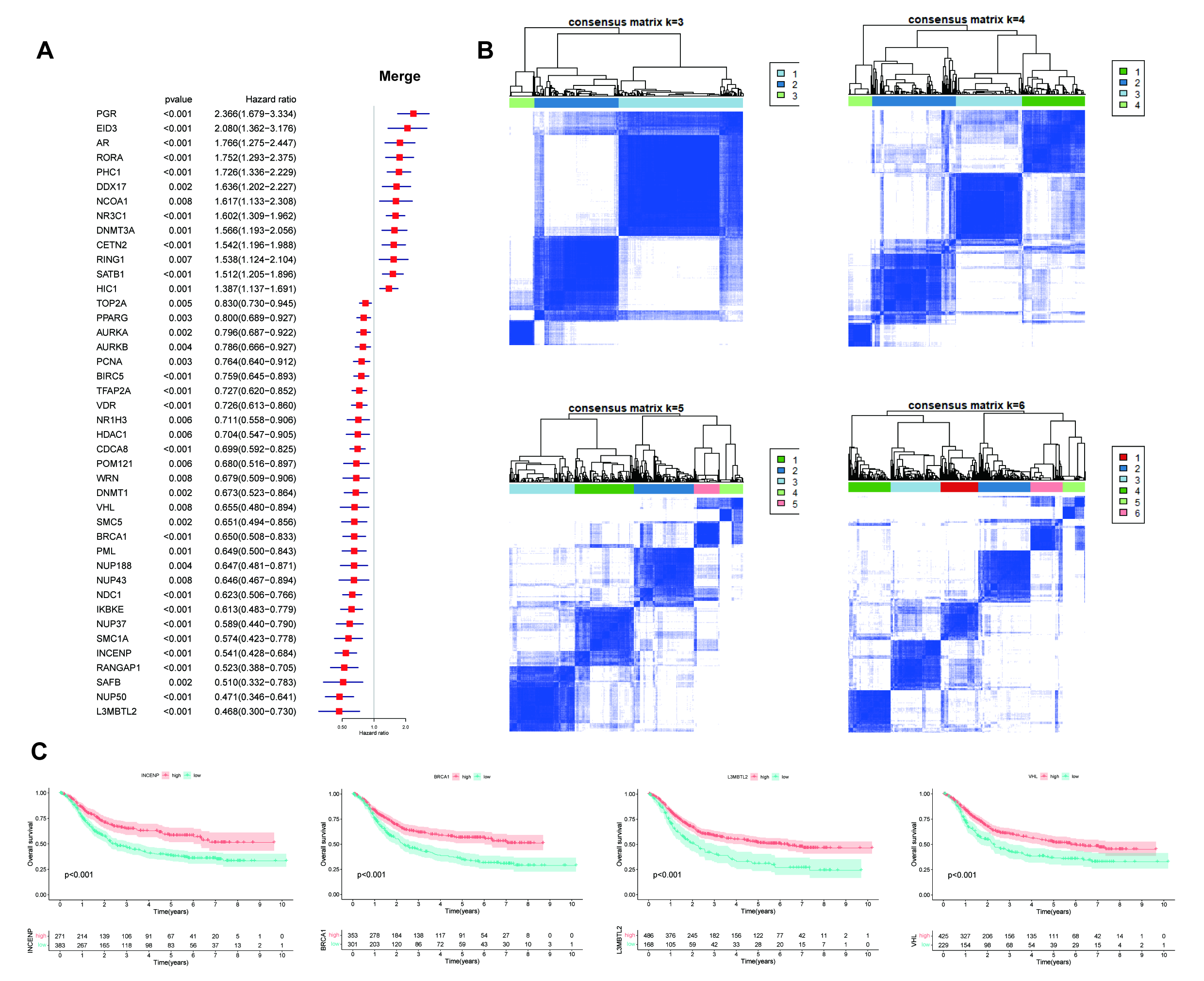

Supplement: Supplementary Figure 1 — Univariate analysis and subtype clustering based on SUMOylation-related genes. (A) Forest plot showing the results of univariate Cox regression analysis for SUMOylation-related genes. (B) Unsupervised clustering analysis dividing samples into distinct molecular subtypes based on SUMOylation-related gene expression (k=3-6). (C) Representive Kaplan-Meier survival curves for favorable SUMOylation-related genes. [file Image1.tif]

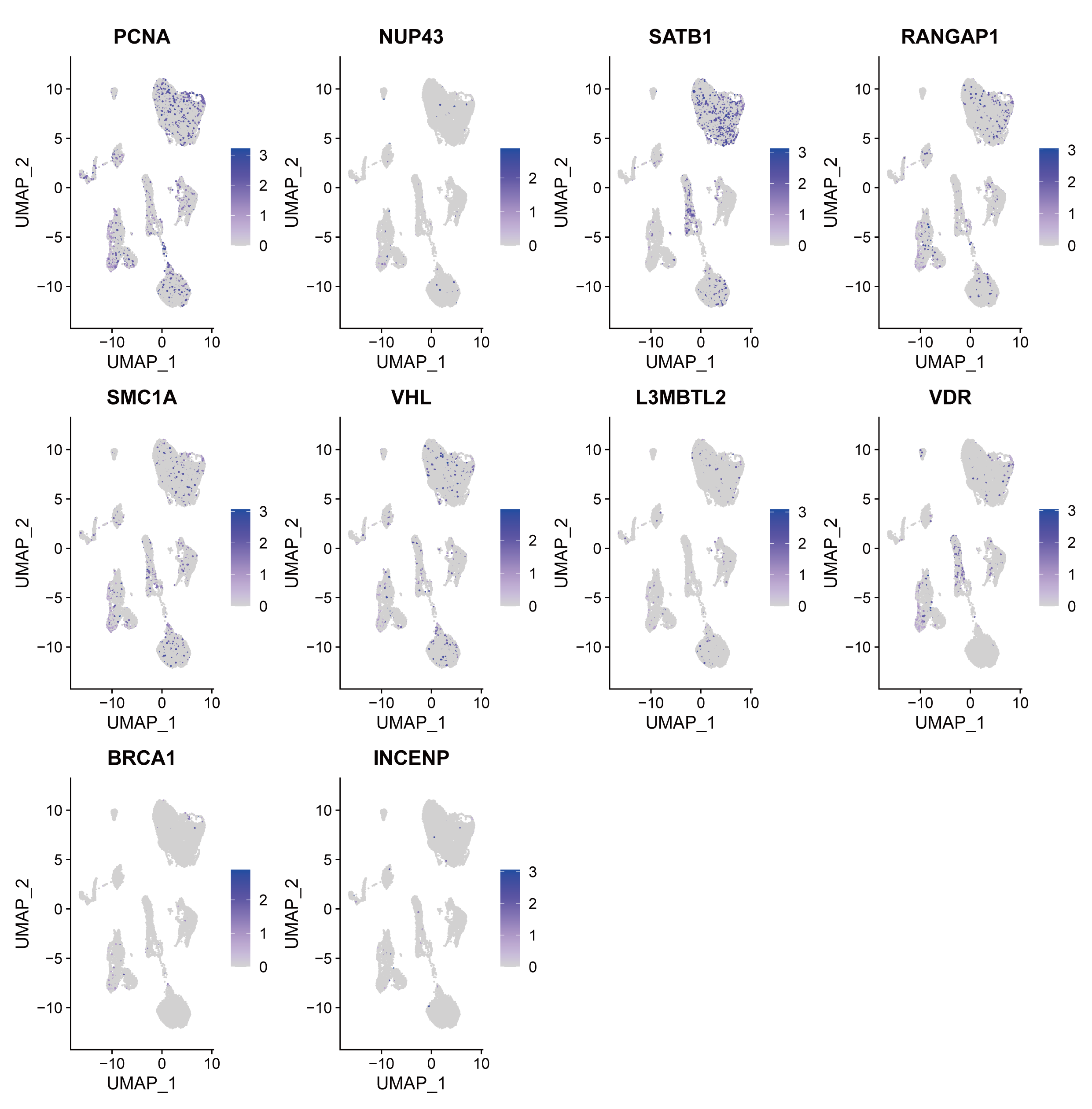

Supplement: Supplementary Figure 2 — Single-cell expression analysis of key genes identified by the random forest model. Uniform Manifold Approximation and Projection (UMAP) plot visualizing the single-cell expression patterns of the 10 key genes identified by the random forest model. [file Image2.tif]

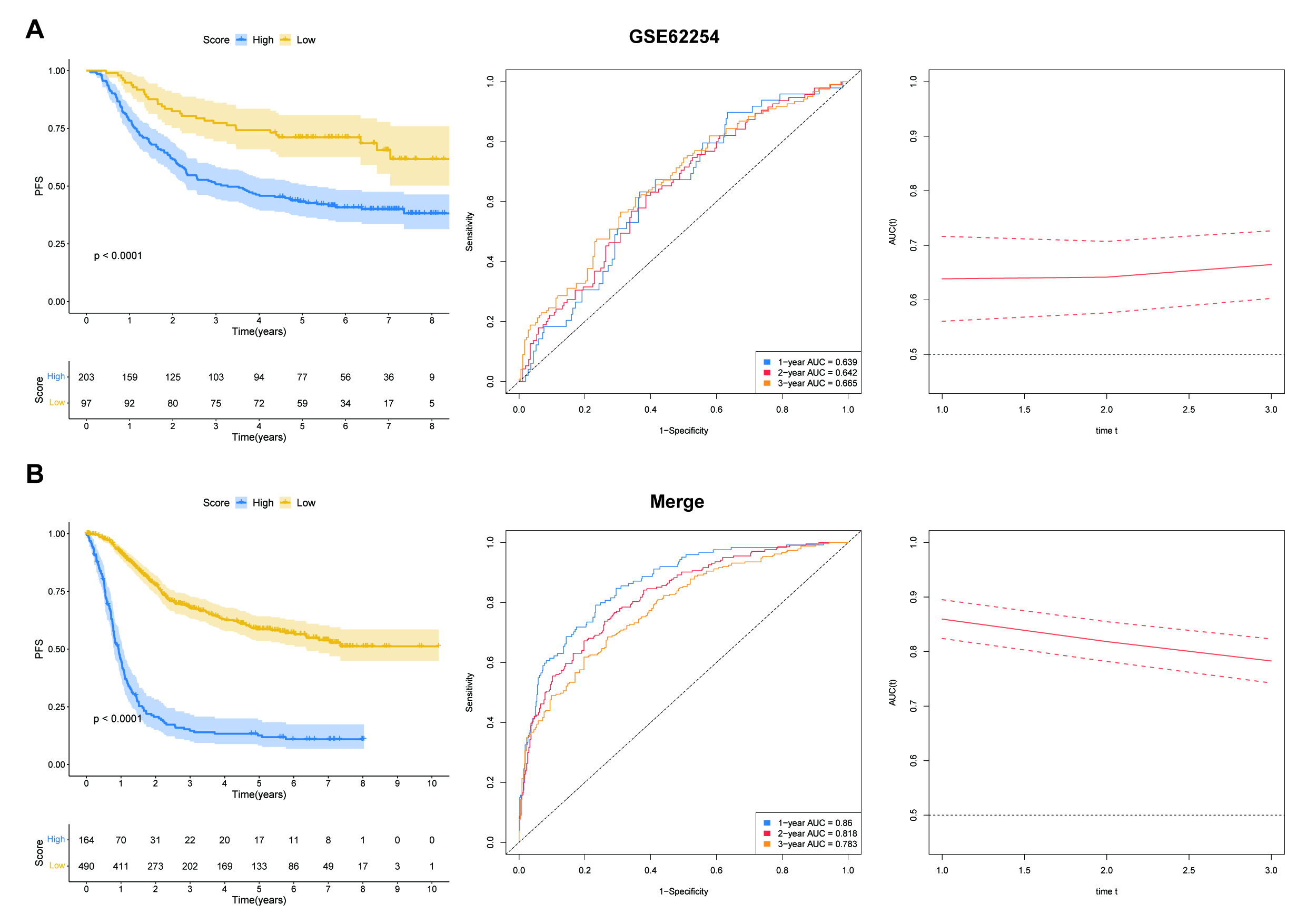

Supplement: Supplementary Figure 3 — Validation of the prognostic model in external cohorts. (A) Validation in the GSE62254 cohort: Left: Kaplan-Meier survival curves stratified by the prognostic model score. Middle: Receiver operating characteristic (ROC) curve evaluating the model’s predictive accuracy. Right: Time-dependent ROC curve assessing the model’s performance at different time points. (B) Validation in the merged cohort, showing consistent performance of the prognostic model. [file Image3.tif]

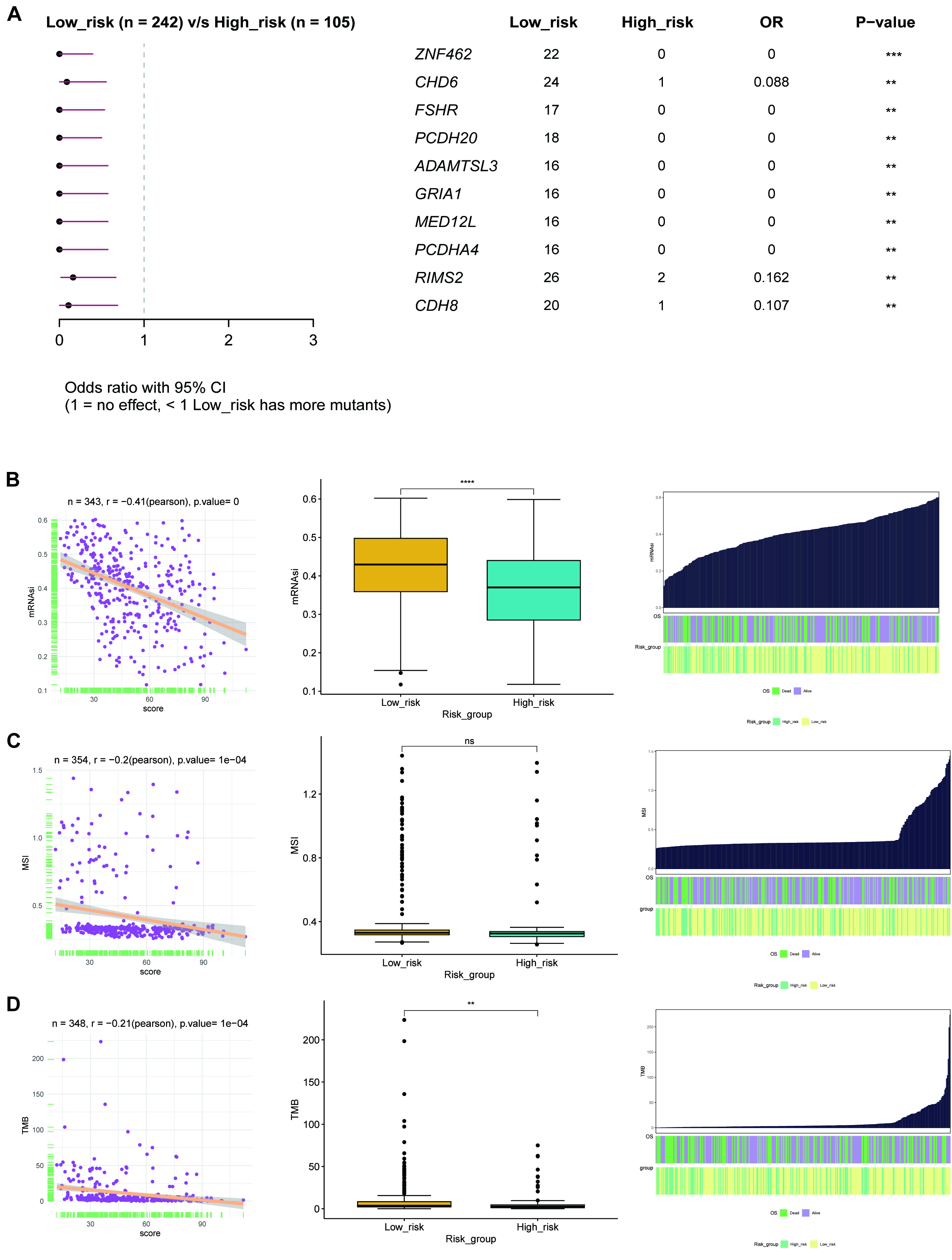

Supplement: Supplementary Figure 4 — Molecular characteristics and immunotherapy response in high- and low-risk groups. (A) Differential gene mutation analysis between high-risk and low-risk groups, highlighting the top mutated genes and their frequencies. (B) Analysis of cell stemness (mRNAsi): Left: Correlation between the prognostic model score and mRNAsi values. Middle: Relationship between the prognostic model score and mRNAsi expression. Right: Distribution of mRNAsi values in high-risk and low-risk groups. (C) Microsatellite instability (MSI) analysis, comparing MSI levels between high-risk and low-risk groups. (D) Tumor mutational burden (TMB) analysis, showing lower TMB in the high-risk group, indicating a potential poor response to immunotherapy. [file Image4.tif]
